# YAP Activation Drives Liver Regeneration after Cholestatic Damage Induced by *Rbpj* Deletion

**DOI:** 10.3390/ijms19123801

**Published:** 2018-11-29

**Authors:** Umesh Tharehalli, Michael Svinarenko, Johann M. Kraus, Silke D. Kühlwein, Robin Szekely, Ute Kiesle, Annika Scheffold, Thomas F.E. Barth, Alexander Kleger, Reinhold Schirmbeck, Hans A. Kestler, Thomas Seufferlein, Franz Oswald, Sarah-Fee Katz, André Lechel

**Affiliations:** 1 Department of Internal Medicine I, Ulm University, 89081 Ulm, Germany; umesh.tharehalli@uni-ulm.de (U.T.); michael.svinarenko@uni-ulm.de (M.S.); Ute.Kiesle@uniklinik-ulm.de (U.K.); alexander.kleger@uni-ulm.de (A.K.); Reinhold.Schirmbeck@uniklinik-ulm.de (R.S.); Thomas.Seufferlein@uniklinik-ulm.de (T.S.); franz.oswald@uni-ulm.de (F.O.); sarah-fee.katz@web.de (S.-F.K.); 2 Medical Systems Biology, Ulm University, 89081 Ulm, Germany; johann.kraus@uni-ulm.de (J.M.K.); silke.kuehlwein@uni-ulm.de (S.D.K.); robin.szekely@uni-ulm.de (R.S.); hans.kestler@uni-ulm.de (H.A.K.); 3 Department of Internal Medicine III, Ulm University, 89081 Ulm, Germany; annika.scheffold@uni-ulm.de; 4 Department of Pathology, Ulm University, 89081 Ulm, Germany; Thomas.Barth@uniklinik-ulm.de

**Keywords:** Notch signalling pathway, cholestasis, YAP activation, Hippo signalling pathway, hepatocyte transdifferentiation, maturation of bile ducts

## Abstract

Liver cholestasis is a chronic liver disease and a major health problem worldwide. Cholestasis is characterised by a decrease in bile flow due to impaired secretion by hepatocytes or by obstruction of bile flow through intra- or extrahepatic bile ducts. Thereby cholestasis can induce ductal proliferation, hepatocyte injury and liver fibrosis. Notch signalling promotes the formation and maturation of bile duct structures. Here we investigated the liver regeneration process in the context of cholestasis induced by disruption of the Notch signalling pathway. Liver-specific deletion of recombination signal binding protein for immunoglobulin kappa j region (*Rbpj*), which represents a key regulator of Notch signalling, induces severe cholestasis through impaired intra-hepatic bile duct (IHBD) maturation, severe necrosis and increased lethality. Deregulation of the biliary compartment and cholestasis are associated with the change of several signalling pathways including a Kyoto Encyclopedia of Genes and Genomes (KEGG) gene set representing the Hippo pathway, further yes-associated protein (YAP) activation and upregulation of SRY (sex determining region Y)-box 9 (SOX9), which is associated with transdifferentiation of hepatocytes. SOX9 upregulation in cholestatic liver injury in vitro is independent of Notch signalling. We could comprehensively address that in vivo *Rbpj* depletion is followed by YAP activation, which influences the transdifferentiation of hepatocytes and thereby contributing to liver regeneration.

## 1. Introduction

Chronic liver disease (CLD) is characterised by a permanent damage of liver cells followed by activation of repair mechanisms and represent a major health problem worldwide. Twenty-nine million people in the European Union suffered from CLD in 2013 [1,2]. Liver transplantation is the only cure for end-stage liver disease patients. Hence, it is of upmost interest to understand the mechanisms controlling liver regeneration in CLD and to identify involved pathways and biomarkers for early diagnosis and treatment. Liver disease affecting bile acid regulation is called cholestatic liver disease. It can be caused by drugs, autoimmune damage of bile ducts, genetic defects and developmental disorders. Liver regeneration in the context of a cholestatic liver disease needs to replace damaged cells, generate branching tubules and a fibrovascular stroma to preserve the new tissue. Several studies addressed the role of Notch signalling in liver development [3,4,5,6,7,8] and liver regeneration [9,10,11]. Notch signalling is an evolutionarily conserved pathway regulating numerous cellular processes including cell fate decision and differentiation [12,13]. Notch is required to commit hepatic progenitor cells (HPC) to the biliary fate and to orchestrate the biliary tree remodelling. Recombination signal binding protein for immunoglobulin kappa j region (RBPJ) is the central downstream effector of Notch signalling and is essential for the activation of the Notch pathway [14]. Liver-specific *Rbpj* deletion in mice causes impaired intrahepatic bile duct development, severe cholestasis, hepatic necrosis and fibrosis [15,16]. Through the capability of a cellular plasticity, the liver is able to generate intra-hepatic bile ducts (IHBD) independent of Notch signalling. Thereby the biliary lineage marker SOX9 is acquired in periportal as well as in interlobular hepatocytes to form an intermediate cell phenotype [16,17].

Here, we show that the liver-specific deletion of *Rbpj* induced growth reduction, liver cholestasis and hepatic necrosis followed by a compensatory liver regeneration. Due to hepatic cholestasis, we depicted increased IQGAP1 expression and subsequent nuclear translocation of YAP, which results in hepatocyte differentiation by induction of SOX9 towards an intermediate phenotype. In addition, from our study of in vitro cholestatic liver injury, we could show that upregulation of *Sox9* expression upon induction of cholestasis is independent of Notch signalling. We suppose the activation of YAP as a mechanism how the cholestatic liver can regenerate upon loss of *Rbpj*.

## 2. Results

### 2.1. Liver-Specific Embryonic Deletion of Rbpj Results in Reduced Body Size and Impaired Survival

**Figure 1:**
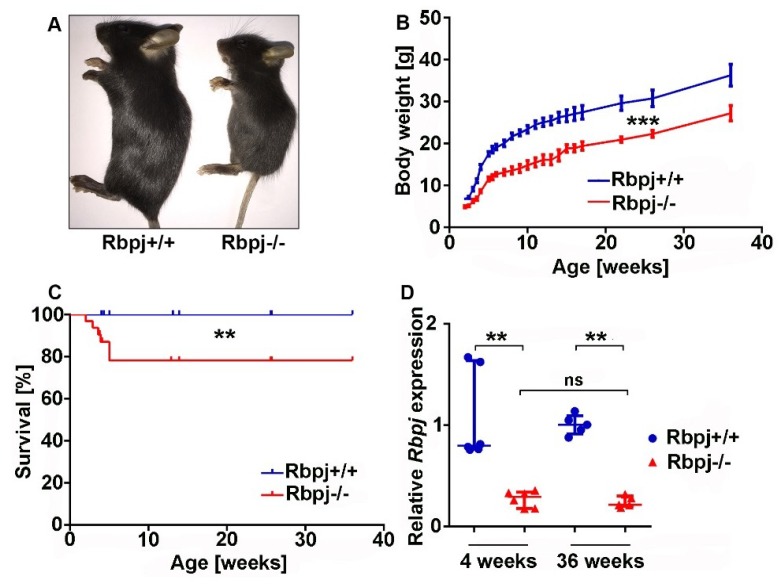
Liver-specific *Rbpj* deletion results in reduced body size and impaired survival. (**A**) Macroscopic photographs of *Rbpj*^+/+^ and *Rbpj*^−/−^ mice at 4 weeks of age (*n* = 5–6). (**B**) Body weight curve of *Rbpj*^+/+^ and *Rbpj*^−/−^ mice, *Rbpj*^−/−^ mice show persistent lower body weight (*n* = 31–32; *** *p* ≤ 0.0001, Pearson coefficient correlation (Two tailed)). (**C**) Survival curve, *Rbpj*^−/−^ mice show impaired survival until 5 weeks of age (*n* = 32; ** *p* ≤ 0.01, Log-rank (Mantel–Cox) test). (**D**) Relative mRNA expression levels of *Rbpj* in 4- and 36-week-old mice. Scatter dot plots showed reduced *Rbpj* expression level in *Rbpj*^−/−^ mice compared to *Rbpj*^+/+^ mice normalised to RNA polymerase II (*n* = 5–6; ** *p* ≤ 0.01, ns = not significant; Mann–Whitney test (Two tailed)).

For the investigation of liver regeneration in the context of cholestatic liver disease, we used a transgenic mouse model for the abrogation of Notch signalling [7]. We crossed a conditional *Rbpj* cKO mouse [18] with an *AlfpCre* mouse [19] on a C57BL/6J background (Figure S1A–C). All *Rbpj* knockout (*Rbpj*^−/−^) mice were phenotypically small (Figure 1A). The body weight was monitored for up to 36 weeks and reduced body weight of *Rbpj*^−/−^ mice was observed at every monitored time point (Figure 1B). The reduced body weight was observed in both sexes and was more pronounced in male mice (Figure S1D,E). Liver-specific deletion of *Rbpj* induces increased lethality within five weeks after birth (Figure 1C). No further lethality was observed at later time point. In order to verify the efficiency of *Rbpj* deletion in the liver, we performed quantitative real-time polymerase chain reaction (qRT-PCR) and Western blotting. We observed reduction of *Rbpj* expression at mRNA and protein levels in *Rbpj*^−/−^ mice (Figure 1D; Figure S1F).

### 2.2. Loss of RBPJ Results in Impaired Maturation of IHBD

**Figure 2:**
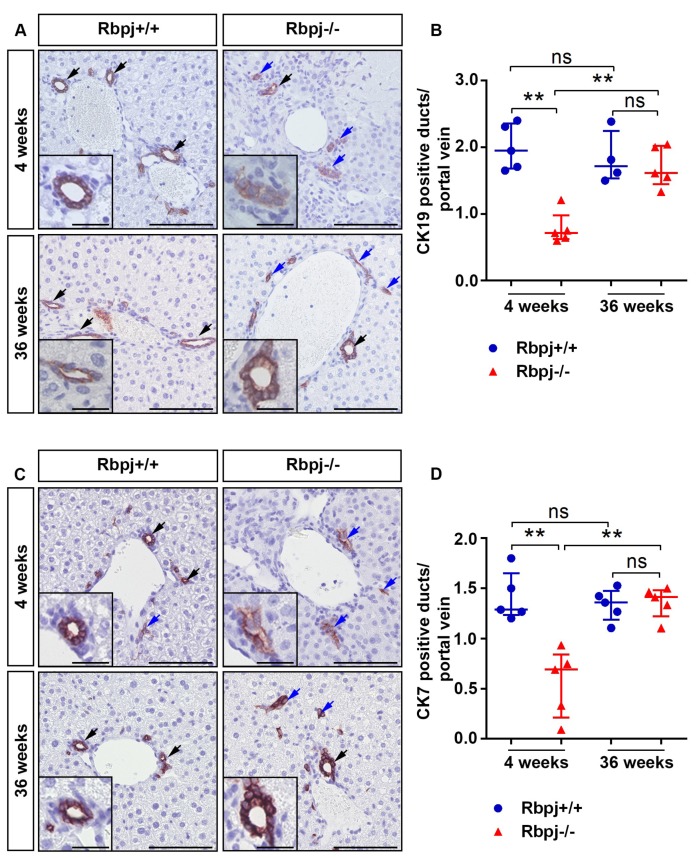
Impaired intrahepatic bile duct development in *Rbpj*^−/−^ mice. Representative photographs of CK19 (**A**) and CK7 (**C**) immunostaining performed on liver sections of 4- and 36-week-old mice. Four-week-old *Rbpj*^−/−^ liver exhibit formation of immature, scattered bile duct structures; there are some clusters of CK19/CK7 positive cells without a clear lumen or dispersed single CK19/CK7 positive cells (blue arrow). In contrast, 36-week-old *Rbpj*^−/−^ liver has a combination of matured CK19/CK7 positive bile duct structures (black arrow) and persisting irregular CK19/CK7 positive clusters (blue arrow) (*n* = 5; scale bar: 100 µm (inlets (A+C) represents a high magnification of a duct structure from the corresponding figure, scale bar: 25 µm)). Scatter dot plots shows CK19 (**B**) and CK7 (**D**) positive lumen structures per portal vein (*n* = 5; ** *p* ≤ 0.01, ns = not significant; Mann–Whitney test (Two tailed)).

Notch signalling is known to regulate cell fate decision and maintenance of stem cells in the liver. In mice, bile duct morphogenesis starts after embryonic day (ED) 15 and continues until the first two weeks after birth. CK19 and CK7 are markers for mature bile ducts, whose expression gradually increases during maturation. We compared *Rbpj*^+/+^ and *Rbpj*^−/−^ mice at the age of 4 and 36 weeks to study IHBD changes (Figure 2A–D). Mature bile ducts were well organized into the mesenchyme around the branches of the portal vein and showed a strong expression of CK19/CK7 in *Rbpj*^+/+^ liver (Figure 2A,C). In sharp contrast, liver from 4-week-old *Rbpj*^−/−^ mice showed CK19/CK7 positive immature tubular structures and cluster of scattered CK19/CK7 positive cells without a lumen indicating impaired maturation of bile duct structures (Figure 2B,D). Interestingly, 36-week-old *Rbpj*^−/−^ mice showed the formation of matured bile duct structures along with persisting immature and scattered biliary epithelial cells (BEC) (Figure 2A,C). No differences were observed in the expression of CK19 mRNA between *Rbpj*^−/−^ and *Rbpj*^+/+^ mice (Figure S2E).

### 2.3. Loss of RBPJ Results in Hepatic Cholestasis and Necrosis

**Figure 3:**
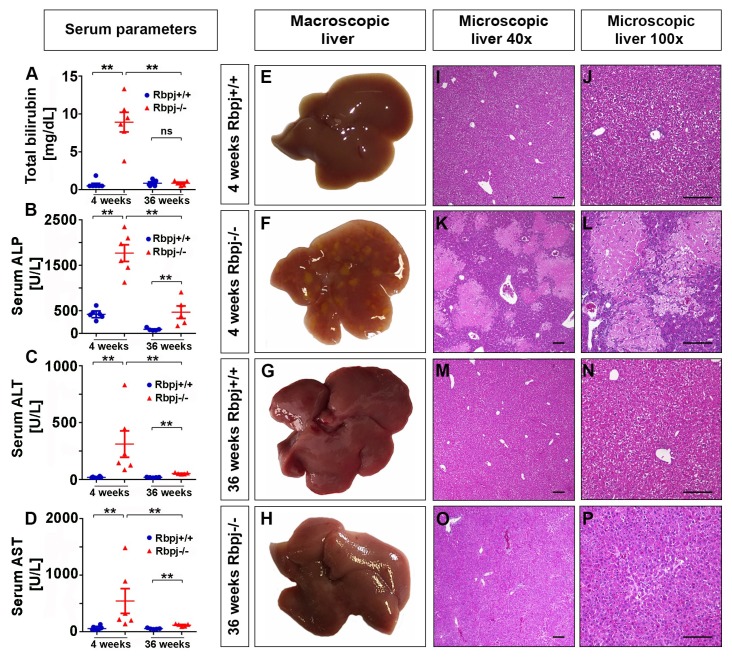
Loss of RBPJ results in cholestasis and hepatic necrosis. (**A**–**D**) Serum parameters indicating liver inflammation and cholestasis. Scatter dot plots of (**A**) TB (total bilirubin (mg/dL)), (**B**) ALP (alkaline phosphatase (U/L)), (**C**) ALT (alanine transaminase (U/L)), and (**D**) AST (aspartate transaminase (U/L)) measured in mouse serum at 4 and 36 weeks from *Rbpj*^+/+^ and *Rbpj*^−/−^ mice (*n* = 5–6; ** *p* ≤ 0.01, Mann–Whitney test (Two tailed)). All four serum parameters are increased in 4-week-old *Rbpj*^−/−^ mice. (**E**–**H**) Representative photographs of macroscopic liver from *Rbpj*^+/+^ and *Rbpj*^−/−^ mice at 4 and 36 weeks (*n* = 5–6). (**I**–**P**) Representative photographs of Hematoxylin & Eosin staining from *Rbpj^+/+^* and *Rbpj*^−/−^ liver. Liver of 4-week-old *Rbpj*^−/−^ mice depicts zone 2 associated confluent necrosis (**K** + **L**), whereas liver from 36-week-old *Rbpj*^−/−^ mice shows recovery from *Rbpj* deletion induced liver necrosis (**O** + **P**) (I = 5–6; scale bar: 100 µm).

Because of impaired IHBD formation through *Rbpj* deletion, mice had developed cholestasis as measured by increased serum parameters total bilirubin (TB), alkaline phosphatase (ALP), alanine aminotransferase (ALT), and aspartate aminotransferase (AST). Interestingly, serum parameters that indicated cholestasis in 4-week-old *Rbpj*^−/−^ mice were significantly reduced in 36-week-old *Rbpj*^−/−^ mice (Figure 3A–D). Histology analysis (Figure 3I–P) in 4-week-old *Rbpj*^−/−^ liver parenchyma showed massive confluent aseptic necrosis within zone 2 (Figure 3F,K,L). Surrounding and infiltrating immune cells as a sign of necrosis were observed at necrotic lesions in the liver of 4-week-old *Rbpj*^−/−^ mice (Figure S2C,D). However, 36-week-old *Rbpj*^−/−^ mice did not show any signs of necrosis and were comparable to *Rbpj*^+/+^ mice, suggesting a proper regeneration of the liver (Figure 3E,G,H–J,M–P and Figure S2B).

### 2.4. Increased Hepatocyte Proliferation and Induction of Hepatocyte Plasticity Due to RBPJ Deficiency-Induced Cholestasis

**Figure 4:**
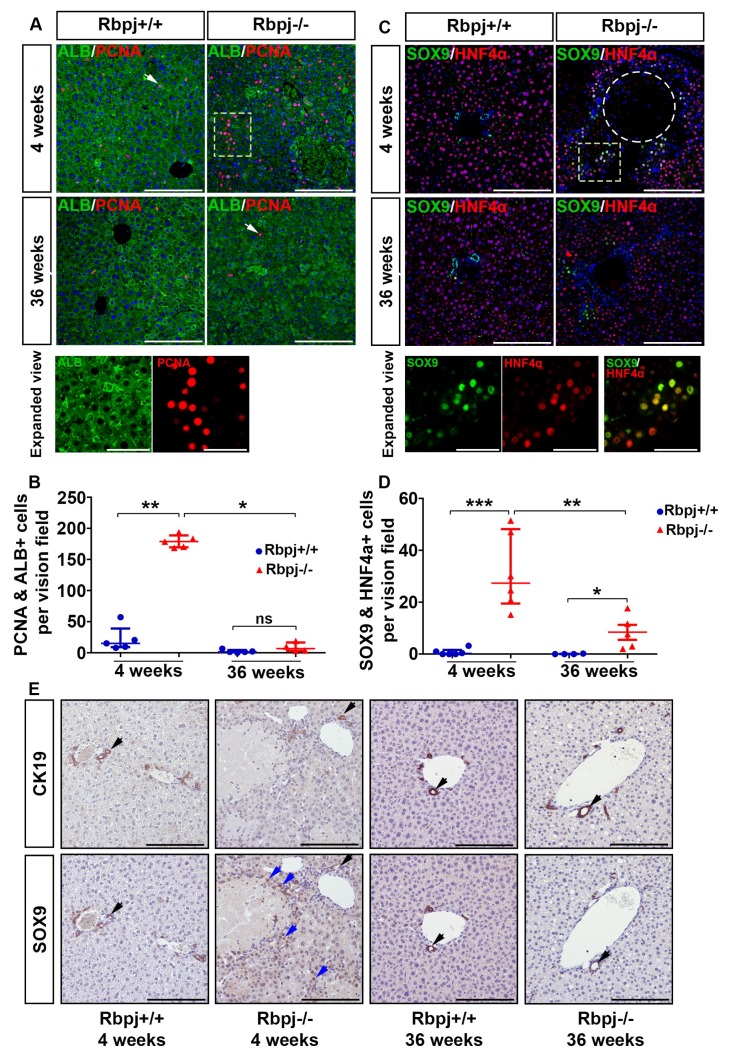
Regenerative proliferation upon RBPJ deficiency induced cholestasis. (**A**) Representative photographs of co-immunostaining of hepatocyte specific marker albumin (green), proliferation marker PCNA (red) and DAPI (blue) of *Rbpj^+/+^* and *Rbpj*^−/−^ liver at the age of 4 and 36 weeks (white arrows indicating proliferating hepatocytes in 4 week Rbpj^+/+^ and 36 week Rbpj^−/−^ liver). Four-week-old *Rbpj*^−/−^ liver show an increase in PCNA^+^ hepatocytes, which does not persist at 36 weeks (*n* = 5; scale bar: 100 µm). The white dotted area of the 4-week-old *Rbpj*^−/−^ liver is shown as an expanded view (scale bar: 25 µm). (**B**) Scatter dot plots of PCNA and albumin double positive cells (*n* = 5; ** *p* ≤ 0.01, * *p* ≤ 0.05, ns = not significant; Mann–Whitney test (Two tailed). (**C**) Representative photographs of co-immunostaining for the hepatic progenitor/cholangiocytic marker SOX9 (green), hepatocyte marker HNF4α (red) and DAPI (blue) (*n* = 5). SOX9 positive hepatocytes surrounding necrotic area indicating induced hepatocyte plasticity upon *Rbpj* deletion induced cholestasis (*n* = 5; scale bar: 100 µm). The dotted circle represents necrotic area. The marked area of the 4-week-old *Rbpj*^−/−^ liver is shown as an expanded view (scale bar: 25 µm). (**D**) Quantification of SOX9 and HNF4α double positive cells in liver from 4 and 36 weeks *Rbpj*^+/+^ and *Rbpj*^−/−^ mice (*n* = 5; *** *p* ≤ 0.001, ** p ≤ 0.01, * *p* ≤ 0.05, Mann–Whitney test (Two tailed)). (**E**) Representative photographs of CK19 and SOX9 immunostaining performed on serial sections (*n* = 5; scale bar: 100 µm). CK19/SOX9 double positive cells (brown staining) are depicted by black arrows, SOX9 single positive cells by blue arrows. SOX9 single positive cells are enriched surrounding the necrotic area (sections are counterstained with hematoxylin which arises as blue stained nuclei).

Impaired IHBD maturation obstructs the bile acid flow, which leads to accumulation of bile acids in the hepatic compartment causing necrosis (Figure 3K,L and Figure S2A). Liver regeneration was observed as a compensatory mechanism for the loss of hepatocytes in 4-week-old *Rbpj*^−/−^ mice as shown by immune histochemistry (IHC) analyses using the proliferation marker PCNA and the hepatocyte marker Albumin (Figure 4A,B). In contrast, livers of 36-week-old *Rbpj*^+/+^ and *Rbpj*^−/−^ mice did not exhibit elevated hepatocyte proliferation indicating a quiescent phenotype (Figure 4A,B). We confirmed these results by western blot analysis for PCNA (Figure S3A). A correlation of the number of PCNA positive hepatocytes with the non-necrotic area revealed that an increased number of hepatocytes were able to proliferate in a less necrotic environment in 4-week-old *Rbpj*^−/−^ mice (Figure S3B). High levels of necrosis suggest an impaired proliferation capacity and an increased mortality of *Rbpj*^−/−^ mice. Since cholangiocyte maturation and differentiation are impaired due to RBPJ deletion, we questioned the possible activation of hepatocyte transdifferentiation as one of the mechanisms for liver regeneration. Under normal physiological conditions, the expression of SOX9 is restricted to bile duct cells and HPCs. SOX9 expression in hepatocytes is a sign of hepatocellular plasticity and enables cells to enter an intermediate phenotype. Therefore, we co-stained liver sections with the progenitor cell marker SOX9 and hepatocyte cell marker HNF4α (Figure 4C,D). In addition, we stained for SOX9 and the biliary epithelial cell marker CK19 on serial sections (Figure 4E). Enrichment of SOX9^+^ HNF4α^+^ and SOX9^+^ CK19^−^ cells surrounding necrotic areas were evident in *Rbpj*^−/−^ liver at the age of 4 weeks (Figure 4C,E). Cellular morphology of SOX9^+^ HNF4α^+^ CK19^−^ cells resembled hepatocytes, indicating the possibility of hepatocyte transdifferentiation following cholestatic liver damage. Our findings are in line with earlier publications reporting activation of SOX9 in cells bearing hepatocyte morphology [16,20]. To estimate proliferating transdifferentiated hepatocytes, we performed SOX9/PCNA co-immunostaining. We observed 13.6% SOX9^+^ proliferating hepatocytes in 4-week-old *Rbpj*^−/−^ mice (Figure S3C,D).

### 2.5. YAP Activation to Bypass Notch Pathway Deficiency

**Figure 5:**
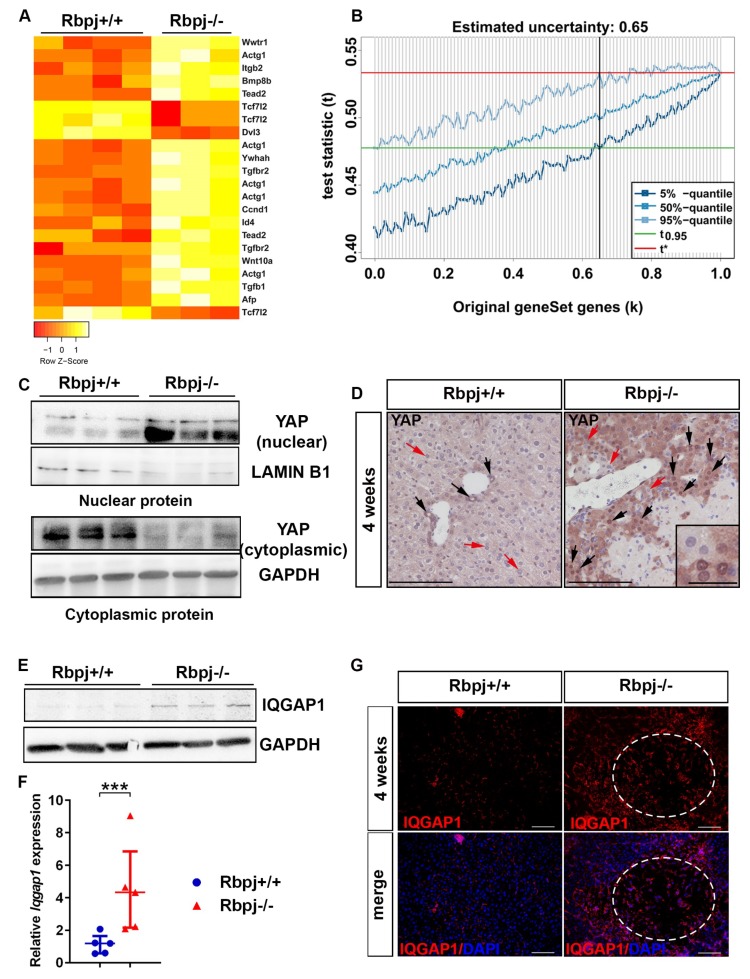
Notch-pathway deficiency induced cholestasis results in YAP activation and deregulation of the Hippo-pathway gene set. (**A**) Heatmap of differentially regulated genes of the Hippo pathway between 4-week-old *Rbpj*^+/+^ and *Rbpj*^−/−^ mice (*n* = 3–4). (**B**) GiANT of the Hippo pathway gene set in 4-week-old murine liver, which quantified the uncertainty in the Hippo pathway (*n* = 3) (t: test statistic of interest; t*: value statistic of the original gene set; k: tested degree of uncertainty). (**C**) Immunoblot of nuclear and cytoplasmic YAP in the liver of *Rbpj*^+/+^ and *Rbpj*^−/−^ mice. (*n* = 3). Full-length immunoblots are depicted in Figure S5A,B (**D**) YAP immunostaining showed increased nuclear staining of YAP in *Rbpj*^−/−^ hepatocytes (*n* = 5; scale bar: 100 µm (inlet shows expanded view of a particular region of the original image, scale bar: 25 µm)); black arrows represent nuclear YAP^+^ cells and red arrow represents nuclear YAP^-^ cells). (**E**) Immunoblot of IQGAP1 of whole liver protein lysates (*n* = 3). Full-length immunoblot is depicted in Figure S5D. (**F**) Relative mRNA expression of *Iqgap1* normalised to RNA polymerase II (*n* = 5; *** *p* ≤ 0.001, Mann–Whitney test (Two tailed)). (**G**) Representative photographs of IQGAP1-IF (red fluorescence signals depict IQGAP1 expression, upper panel) and IQGAP1-IF with nuclear DAPI stain (lower panel, merged) of *Rbpj*^+/+^ and *Rbpj*^−/−^ liver. Necrotic area is circled by white dots (*n* = 3; scale bar: 100 µm).

We performed microarray analyses of whole liver tissue from 4-week-old *Rbpj*^+/+^ and *Rbpj*^−/−^ mice to identify major regulatory pathways and dysregulated genes in the cholestatic liver due to loss of *Rbpj* (Tables S1 and S2), which influence hepatocyte plasticity and/or contribute to liver regeneration. Interestingly, distinct pathways such as metabolic pathways, Hippo and PI3K-AKT were dysregulated due to *Rbpj* deletion (Table S1). We focused on the Hippo signalling pathway, which is the guardian of organ size regulation, involved in cell fate decision [21]. According to the Kyoto Encyclopedia of Genes and Genomes (KEGG) database, 156 genes are associated to the Hippo signalling pathway (Table S3). The 14 genes of the Hippo signalling pathway presented by 22 oligonucleotides on the microarray were differentially regulated upon *Rbpj* deletion, including Hippo pathway regulators, YAP target genes and increased expression of the YAP paralogue *Taz* (*Wwtr1*) (Figure 5A). A gene set enrichment analyses by using the partial resampling approach GiANT [22] and also conventional Gene Set Enrichment Analysis (GSEA) [23,24] revealed a robust enrichment of the Hippo signalling pathway (Figure 5B, Figure S4A).

To look for Hippo pathway involvement in human patients with cholestatic diseases, we reanalysed a recently published dataset of infants with biliary atresia (BA) and non-BA [25]. BA is a neonatal cholestatic disease characterised by a progressive fibro-inflammatory obstruction of the extrahepatic bile ducts. Infants with BA and non-BA were analysed for a Hippo pathway gene set as well as normal controls from the same study [25]. Enrichment of the Hippo pathway gene set was also evident after reanalysis of expression data of BA vs. control and non-BA vs. control (Figure S4B,C).

In the quiescent liver, YAP is constantly phosphorylated and degraded in the cytoplasm and thereby regulates the organ size [26]. During liver injury or after partial hepatectomy, non-phosphorylated YAP translocate to the nucleus and activates cell proliferation [27,28]. We analysed the localization of YAP by western blot analysis (Figure 5C) and IHC (Figure 5D). We could detect an increased YAP level in the nucleus and a decreased level in the cytoplasm in 4-week-old *Rbpj*^−/−^ mice (Figure 5C). In addition, we analysed the expression of Hippo pathway regulators *Yap*, *Tead2* and *Yap* target genes such as *Afp*, *Itgb2, Gpc3, Ctgf, Cyr61 and Ankrd1* by qRT-PCR analysis. Along with an increased nuclear level of YAP (Figure 5C, Figure S5A,B), we found an upregulation of *Tead2*, *Afp*, *Itgb2, Gpc3, Ctgf and Ankrd1* in *Rbpj*^−/−^ liver (Figure S5C). We did not observe changes in the mRNA expression of *Cyr61*and *Yap* (Figure S5C).

The scaffolding protein IQGAP1 is claimed to be a key mediator in the process of YAP activation induced by elevated bile acids [29]. IQGAP1 is upregulated under cholestatic conditions to regulate nuclear translocation of YAP. Patients with a variety of biliary dysfunctions exhibit enhanced IQGAP1 and nuclear YAP expression. It was shown, that a disruption of the nuclear receptors farnesoid X receptor (FXR) and small heterodimer partner (SHP) led to elevated bile acid levels [29]. In addition, increased Ki67 levels are reported upon IQGAP1 overexpression whereas Ki67 positive cells were reduced in *Iqgap1* knockout mice upon CA diet which induces cholestasis [29]. In line with that report, *Iqgap1* is upregulated in 4-week-old *Rbpj*^−/−^ mice (Figure 5E,F, Figure S5D). Immunofluorescence (IF) analysis showed enhanced level of IQGAP1 positive non-hepatocytes and a small fraction of IQGAP1^+^ hepatocytes in the liver of *Rbpj*^−/−^ mice (Figure 5G). In correlation with an increased YAP activation we observed a higher liver weight to body weight ratio in 4-week-old *Rbpj*^−/−^ mice (Figure S5E).

### 2.6. Induction of Cholestatic Liver Injury In Vitro Induces Sox9 and Hes1 Expression Independent of Notch Signalling

**Figure 6:**
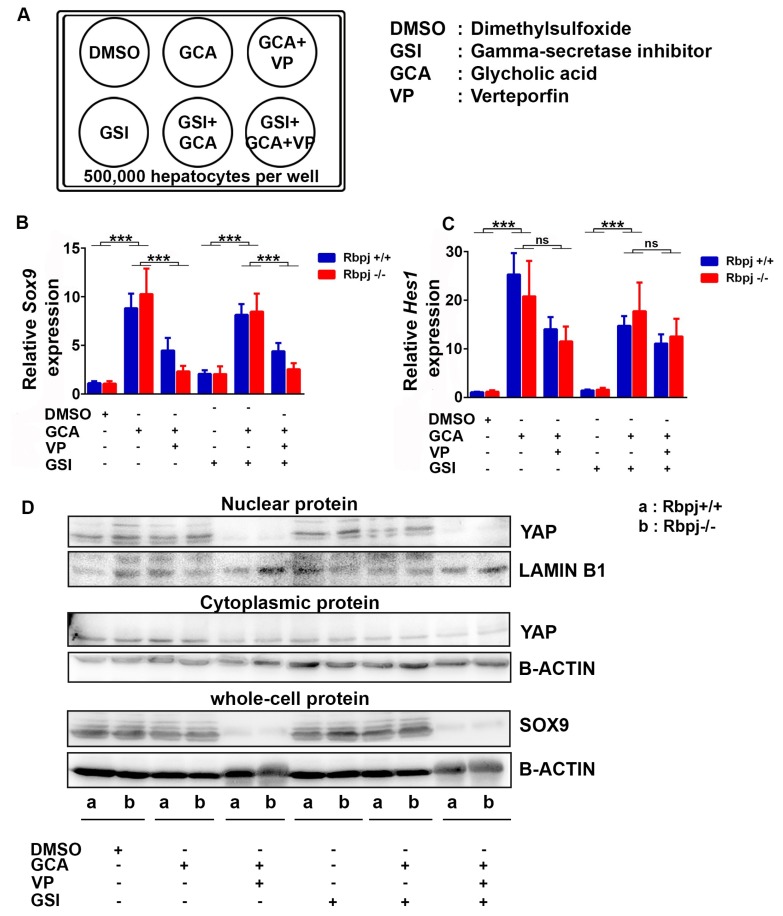
Cholestatic liver injury in vitro results in *Sox9* upregulation independent of Notch signalling. (**A**) Scheme for in vitro treatment of hepatocytes isolated from *Rbpj*^+/+^ and *Rbpj*^−/−^ mice. (**B** + **C**) Bar chart showing relative mRNA expression of *Sox9* (**B**) and *Hes1* (**C**) normalised to RNA polymerase II (*n* = 5–7; *** *p* ≤ 0.001, ns = not significant; Mann–Whitney test (Two tailed)). (**D**) Immunoblot of nuclear YAP, cytoplasmic YAP and SOX9 from in vitro treated primary hepatocytes isolated from *Rbpj^+/+^* and *Rbpj*^−/−^ mice. Full-length blot for SOX9 is depicted in Figure S6E.

In the *Rbpj*^−/−^ mouse model, we observed transdifferentiation of hepatocytes by SOX9 upregulation. We assume that YAP activation has a major role in hepatocyte transdifferentiation upon induced cholestasis. To prove our hypothesis, we treated primary hepatocytes from *AlbCre^+^ Rbpj*^+/+^ and *AlbCre^+^ Rbpj*^−/−^ mice with glycocholic acid (GCA) to mimic cholestatic liver injury in vitro [30] (Figure 6A). Since the *AlfpCre* mediated deletion of *Rbpj* had a severe phenotype, we used a different Cre driver line for this experiment. *AlbCre^+^ Rbpj*^−/−^ mice did not show lethality, we observed a normal body size, no increase in serum level of liver enzymes and rarely hepatic necrosis (Figure S6A–D). In parallel, we treated the hepatocytes with verteporfin (VP) to block YAP/TEAD-dependent transcription [31,32] and a gamma-secretase inhibitor IX (GSI-IX) to block Notch signalling in combination with GCA. The co-treatment of GCA and VP gradually reduced expression levels of *Sox9* and the YAP target genes *Cyr61, Ctgf, Afp and Ankrd1* (Figure 6B,D, Figure S6E,F) indicating a direct link between cholestasis mediated expression of *Sox9* through activation of YAP, which is independent of RBPJ (Figure 5C). The upregulation of *Sox9* by GCA was limited to the RNA level, whereas inhibition of *Sox9* under GCA + VP treatment was present on RNA and protein level (Figure 6B,D). In addition, SOX9 has been reported to regulate expression of *Hes1* and can guide the differentiation of dedifferentiated hepatocytes to biliary epithelial cells (BEC) [33]. We observed increased *Hes1* expression level upon GCA treatment and a decreased *Hes1* expression level upon blockade of YAP activation (Figure 6C). In addition, we monitored a Notch-independent regulation of *Sox9* and *Hes1* in in vitro cultivated hepatocytes. GSI-IX treatment which blocks Notch signalling did not result in additional changes between *Sox9* and *Hes1* expression (Figure 6B,C). In our experimental setup, we could show by using *Rbpj*^−/−^ hepatocytes and by GSI-IX treatment, that the regulation of *Sox9* and *Hes1* is independent of intact Notch signalling.

### 2.7. Chronic Liver Damage Subsequently Induced by the Loss of RBPJ Results in Liver Fibrosis

**Figure 7:**
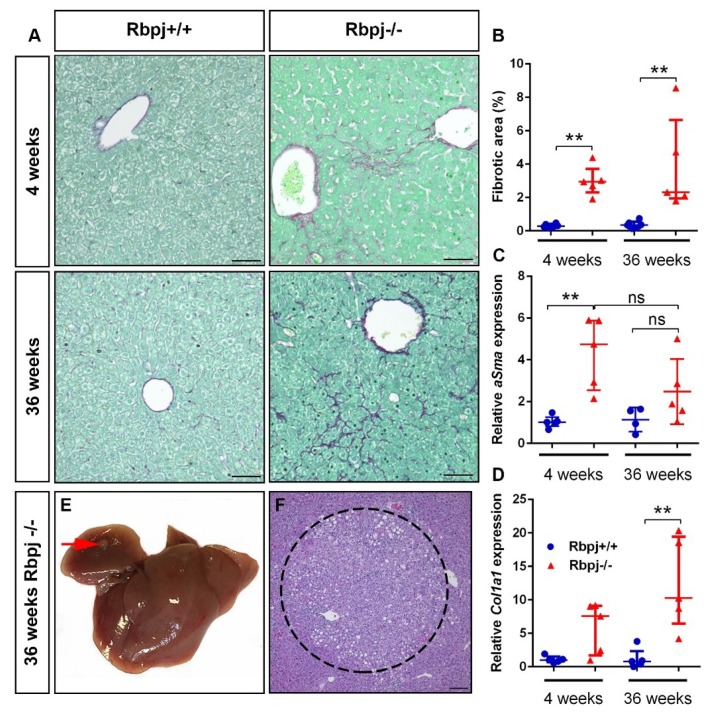
Liver-specific conditional deletion of *Rbpj* results in liver fibrosis. (**A**) Representative photographs of picro-sirius red staining on liver sections from *Rbpj*^+/+^ and *Rbpj*^−/−^ mice at 4 and 36 weeks of age. (*n* = 5–6; scale bar: 100 µm). (**B**) Scatter dot plots showing quantification of fibrotic area determined by picro-sirius red staining (*n* = 5–6, ** *p* ≤ 0.01, Mann–Whitney test (two tailed)). (**C** + **D**) Relative mRNA expression of *αSma* (**C**) and *Col1a1* (**D**) normalised to RNA polymerase II (*n* = 5–6; ** *p* ≤ 0.01, ns = not significant; Mann–Whitney test (two tailed)). (**E** + **F**) Macroscopic dysplastic nodule (**E**) of 36-week-old *Rbpj*^−/−^ liver (red arrow represents macroscopic liver foci) and (**F**) corresponding histology, dysplastic nodule is circled by black dots (scale bar: 100 µm).

The observed phenotype of cholestasis and hepatic necrosis in the *Rpbj*^−/−^ mouse model reflects CLD. CLD is a well-known cause of liver fibrosis and hepatocarcinogenesis. Therefore, we carefully analysed the samples for liver fibrosis and signs of tumor formation. It has been shown that Notch activation/repression, as well as loss of *Rbpj*, is involved in tumor formation [34,35,36]. Here we depicted that liver-specific loss of *Rbpj* resulted in liver fibrosis, which is slightly increased in 36-week-old *Rbpj*^−/−^ mice (Figure 7A,B). The micronodular fibrosis was located in the periphery of the necrotic area in 4-week-old *Rbpj*^−/−^ mice. In 36-week-old *Rbpj*^−/−^ mice, the fibrosis persisted in zone 2, in addition, an increase of fibrosis was seen around the central lobular veins. We observed by qPCR analysis an increased expression of *αSma*, a marker for stellate cell activation (Figure 7C) and *Col1a1*, a marker for liver fibrosis (Figure 7D) in *Rbpj*^−/−^ mice. We could detect liver foci formation in one out of seven *Rbpj*^−/−^ mice at the age of 36 weeks (Figure 7E,F) suggesting that increased liver fibrosis formation by the loss of functional RBPJ might play a role in hepatocarcinogenesis.

## 3. Discussion

The current study provides experimental evidence that YAP activation upon *Rbpj* deletion induced cholestasis is an important mechanism in liver regeneration. The biliary phenotype, which we report in our study is reminiscent of that observed in transgenic mouse models with inactivation of HES1, JAGGED1, RBPJ, or LKB1 [4,7,37,38]. These studies analysed the role of Notch signalling during liver development, on biliary differentiation and morphogenesis. *Rbpj* cKO mice were used in combination with different Cre recombinase driver lines to achieve liver-specific deletion of *Rbpj* [7,8,11,15,16,17]. Two of the most commonly used Cre lines are the *AlbCre* [39] and the *AlfpCre* transgenic mouse line [20]. Notch signalling was shown to control liver development by regulating the biliary differentiation and the three-dimensional architecture of intrahepatic bile ducts [8]. Disruption of Notch signalling leads to defects in the communicating intrahepatic bile duct network [7]. In addition, it was shown in *AlbCre^+^ Rbpj*^−/−^ mice that the intrahepatic bile duct regeneration does not require Notch signalling [16]. We focused our study on 4- and 36-week-old mice to address molecular mechanisms involved in the regenerative process of the liver.

The impaired life span of *Rbpj*^−/−^ mice is due to severe cholestasis and massive necrosis, which reduces the regenerative potential in around one-third of the mice to a life-threatening event. The severity of induced necrosis is 8.2-fold higher in the *AlfpCre^+^ Rbpj*^−/−^ compared to the *AlbCre^+^ Rbpj*^−/−^. By use of the *AlbCre* line the mice did not show growth retardation and impaired survival. Necrosis was rarely detectable in 4-week-old *AlbCre^+^ Rbpj*^−/−^ mice. The Cre-recombinase is induced at ED16.5 under the *Alb* promotor whereas the *Alfp* promotor is active before ED10.5 [20], which leads to an earlier onset of *Rbpj* deletion and result in a more severe phenotype. We observed the growth defect at the suckling/weaning transition, which correlates with an increase in lethality. The severity of necrosis correlates with an impaired regeneration potential which was not reported before.

It is still under debate which mechanisms are involved in liver regeneration to restore the liver cell mass and to maintain liver homeostasis in the context of a cholestatic liver. This study shows that *Rbpj* deletion results in severe cholestasis measured by increased levels of TB, ALP, ALT and AST, and thereby leads to hepatic necrosis. The importance of the Notch signalling pathway has been linked to diseases like BA and Alagille syndrome, which are two rare cholestatic diseases during early childhood [40,41,42,43,44,45,46]. We found that the Hippo pathway enrichment signature by GiANT analysis in human disease by comparing BA and non-BA to control patients suggesting the Hippo pathway might be important in the regeneration process of neonatal cholestasis.

The liver is characterised by a cellular plasticity, which is important for liver regeneration upon liver injury [47,48]. It was reported in *AlbCre^+^ Rbpj*^−/−^ mice that *Sox9* mRNA levels decreases after *Rbpj* deletion at P3 but was similar at P60 compared to wildtype mice [15]. In the same mouse model, another group reported increased SOX9 level by IHC at P30 which disappeared by P60 [11]. Both studies reported about parenchymal necrosis but without a correlation to regeneration. The necrosis was not significant in *Rbpj*^−/−^ mice [15]. In our study, we observed SOX9^+^ cells at 4 weeks and at a reduced, but still significant, level at 36 weeks of age in *Rbpj*^−/−^ mice. We observed in the liver of *Rbpj*^−/−^ mice HNF4α^+^ cells with hepatocyte morphology, carrying the ductal and progenitor marker SOX9. SOX9^+^ CK19^−^ cells were enriched in 4-week-old *Rbpj*^−/−^ mice. The occurrence of these intermediate cells positive for HNF4α and SOX9 but negative for CK19 links to the regeneration process of hepatocyte transdifferentiation rather than activation of HPCs or hepatogenic differentiation of mesenchymal stem/progenitor cells which was reported in human liver disease specimens [49].

Hepatocytes and in higher amount non-hepatocytes expressed the scaffolding protein IQGAP1 which was highly increased in the liver of 4-week-old *Rbpj*^−/−^ mice. Increased bile acid levels were shown in a transgenic mouse model with a defect in bile acid homeostasis. Deletion of the nuclear receptors FXR and SHP resulted in increased IQGAP1 levels, nuclear YAP expression and liver carcinogenesis, indicating that the accumulation of bile acids leads to a nuclear YAP translocation by activating a pathway which is dependent on the induction of IQGAP1 [29]. YAP is a key player in Hippo signalling pathway and is known to be an important regulator of organ size, cell fate and to be involved in carcinogenesis [21]. However, the knockout of YAP leads to impaired liver regeneration in the cholestatic liver [27]. In addition, increased IQGAP1 and nuclear YAP localisation have been reported in human biliary disorders and bile duct ligated mice, a model for experimentally induced cholestasis [27,29]. The current study depicts YAP activation as an essential mechanism for liver regeneration upon *Rbpj* loss induced cholestasis. Thereby, initiate a program for hepatocyte transdifferentiation, which involves upregulation of SOX9.

We performed in vitro studies by using freshly isolated hepatocytes to study the importance of Notch signalling upon cholestatic liver injury in vitro. Interestingly, we could show that Notch depletion either by the use of *AlbCre^+^ Rbpj*^−/−^ hepatocytes or by GSI-IX treatment on *AlbCre^+^ Rbpj^+/+^* hepatocytes, which blocks Notch signalling, does not influence SOX9 expression in cholestatic liver injury in vitro. GCA treatment of hepatocytes from *AlbCre^+^ Rbpj^+/+^* and *AlbCre^+^ Rbpj*^−/−^ mice mimics a cholestatic liver disease in both genotypes and results in an increase of *Sox9* mRNA level. We do not see differences in SOX9 protein levels. This might occur due to the fact that in vitro cultivated primary hepatocytes are quiescent and do not undergo cell division [50]. However, SOX9 expression was clearly blocked by the YAP inhibitor VP. This describes the activation of SOX9 via YAP activation, as a Notch-independent mechanism in cholestatic liver injury in vitro. In contrast to the in vitro model, the in vivo mouse model depicted a cholestatic liver disease only in *Rbpj*^−/−^ mice due to impaired IHBD maturation, *Rbpj^+/+^* mice did not develop a cholestatic liver disease.

As reported above, we observed severe cholestasis in *Rbpj*^−/−^ mice. Severe cholestasis generates a chronic damage of the liver, leading to activation of macrophages for the clearance of necrotic areas. This might also stimulate fibrosis formation and, thereby, could be a risk factor for the initiation of hepatocarcinogenesis in the absence of Notch signalling. Hepatic stellate cells are able to orchestrate the clearance of necrotic cells by conversion of Kupffer cells to M1-like proinflammatory macrophages, which increases phagocytic activity [51]. Here we showed that deletion of *Rbpj* leads to liver fibrosis, which slightly increases, with age and the formation of liver foci in one mouse. So far, the importance of Notch signalling in hepatocarcinogenesis is addressed by overexpression of Notch pathway components [34,52,53,54] and by repression of Notch signalling, which was identified in an interacting network of Hippo/Wnt/β-catenin/Notch signalling [35]. However, the work of Kulic et al. showed loss of RBPJ in human cancer and cancer cell lines, but only one liver cancer cell line was analysed [36]. Future work is needed to address the importance of RBPJ loss in hepatocarcinogenesis.

In conclusion, we suppose the YAP activation as a mechanism for a compensatory liver regeneration after RBPJ ablation induced cholestasis. The liver-specific deletion of *Rbpj* leads to loss of Notch signalling, which results in impaired IHBD formation and, thereby, an accumulation of bile acid in liver leading to cholestasis. The accumulation of bile acid generates an increased expression of the scaffolding protein IQGAP1, which facilitates nuclear YAP translocation to trigger liver regeneration. We claim this mechanism as an important driver for liver regeneration upon RBPJ loss-induced cholestasis.

## 4. Materials and Methods

### 4.1. Mouse Model

*Rbpj*^flox/flox^ mice [18] were crossed with *AlfpCre* mice [19] to generate liver-specific *Rbpj* knockout mice. The following cohorts were generated and utilized in this study: *AlfpCre*^+^ *Rbpj*^flox/flox^ (*Rbpj*^−/−^) and *AlfpCre*^−^ *Rbpj*^flox/flox^ (*Rbpj*^+/+^). Hepatocytes used for in vitro studies were isolated from *AlbCre*^+^ *Rbpj*^flox/flox^ and *AlbCre*^−^ *Rbpj*^flox/flox^ mice [34]. The mice were maintained in a specific pathogen-free environment. All mice received human care and study protocols comply with the institution’s guidelines (Animal Research Centre of Ulm University). The state government of Baden-Württemberg (protocol number 35/9185.81-3/1259; date of approval 8 March 2016) approved all animal experiments.

### 4.2. Liver Histology

Mouse liver tissue was collected and incubated in 4% paraformaldehyde (PFA) for 16 h, processed through ethanol and xylene series, and embedded in paraffin. Sections 3.5 µm thick were used for hematoxylin and eosin (H&E) staining. The necrotic area was calculated by measuring the area of individual necrotic spots per vision field (100×) using the ImageJ software (available online: https://imagej.nih.gov/ij/). In total 10 vision fields per liver were analysed (*n* = 5–6 mice).

### 4.3. Immunostaining

Paraffin-embedded mouse liver tissues were sectioned at 5 µm thickness and used for IHC or IF staining. Antigen unmasking was performed using antigen-unmasking solution (Vector Laboratories, Burlingame, CA, USA) in a steamer for 35 min. Staining was performed with the appropriate primary antibody (Table S4) incubation overnight at 4 °C, followed by corresponding HRP-labelled secondary antibody incubation for 1 h at room temperature (RT). Nova-red (Vector Laboratories, Burlingame, CA, USA) was used for developing chromogenic staining and sections were counterstained with 20% hematoxylin. For immune fluorescence staining, sections were processed similarly to chromogenic staining. After incubation with a corresponding fluorescence-conjugated secondary antibody for 1 h at RT, the nucleus was counterstained with DAPI (Sigma-Aldrich, St Louis, MO, USA).

### 4.4. Protein Isolation and Western Blotting

For whole cell protein isolation, liver tissue or primary hepatocytes were homogenised and lysed in 1× RIPA buffer (50 mM TrisHCl pH 8, 150 mM, 1% NP-40, 0.5% deoxycholate, 0.1% SDS, 1 mM NaVO_3_, 1 mM DTT, 1 mM PMSF) containing protease inhibitor cocktail solution. Protein lysates were stored at −80 °C until analysed. For nuclear and cytoplasmic protein isolation, liver tissue was homogenised using Dignam A lysis buffer (10 mM HEPES pH 7.9, 1.5 mM MgCl_2_, 10 mM KCL, 50 mM PMSF, 1 M DTT and protease inhibitor) and centrifuged. The supernatant and the pellet were collected separately. The supernatant consists the cytoplasmic protein fraction, which was stored at −80 °C. For nuclear protein fraction, the pellet was washed in Dignam B buffer (Dignam A with 0.1% Triton X-100) and afterwards re-suspended in Dignam C buffer (20 mM HEPES pH 7.9, 25% glycerol, 0.42 M NaCl, 1.5 mM MgCl_2_ and 0.2 mM EDTA, 1 mM DTT, 1 mM PMSF, 1 mM Na_3_VO_4_ and protease inhibitor) and stored at −80 °C until analysed. Bradford assay was used to measure protein concentration. Standard western blotting protocol was adopted for western blot experiment.

### 4.5. RNA Isolation

Total RNA was isolated from liver tissue using the RNeasy Mini Kit (Qiagen, Valencia, CA, USA) according to the manufacturer’s guidelines and the quality of RNA was determined using the 2100 Bioanalyzer (Agilent Technologies, Santa Clara, CA, USA). RNA samples with a RIN (RNA integrity number) value above 7.0 were used in this study.

### 4.6. Quantitative Real-Time PCR (qRT-PCR)

cDNA was synthesised from total RNA using the Reverse Transcription System (Promega, Madison, WI, USA) according to the manufacturer’s guidelines. cDNA was amplified by using iTaq Universal SYBR Green Supermix (BIO-RAD, Hercules, CA, USA) in a total volume of 10 µL. Used primer are listed in Table S5.

### 4.7. Serum Parameters

Liver-specific serum enzymes such as ALT, AST, ALP and TB were measured using Reflotron test stripes (ROCHE, Penzberg, Germany).

### 4.8. Gene Expression Analysis

Gene expression analysis was carried out using the SurePrint G3 Mouse Gene Expression 8x60K Microarray Kit (Design ID 028005; Agilent Technologies, Santa Clara, CA, USA). Samples were labeled with the Low Input Quick Amp Labeling Kit (Agilent Technologies, Santa Clara, CA, USA) according to the manufacturer’s guidelines. Slides were scanned using a G2565CA microarray scanner (Agilent Technologies, Santa Clara, CA, USA). Expression data were extracted using the Feature Extraction software (Agilent Technologies, Santa Clara, CA, USA). All expression data were deposited in Gene Expression Omnibus (GEO accession number GSE121302). Pre-processing of expression data was performed according to Agilent’s standard workflow. Using 5 quality flags (gIsPosAndSignif, gIsFeatNonUnifOL, gIsWellAboveBG, gIsSaturated, and gIsFeatPopnOL) from the Feature Extraction software output (Agilent Technologies, Santa Clara, CA, USA), probes were labeled as detected, not detected, or compromised. Gene expression levels were background corrected, and signals for duplicated probes were summarized by geometric mean of non-compromised probes. After log2 transformation, a percentile shift normalization at the 75% level and a baseline shift to the median baseline of all probes was performed. Differentially expressed genes were calculated based on shrinkage-T statistic (false discovery rate < 0.1). Pathway enrichment analysis (KEGG pathways) was calculated by Fisher’s exact test (false discovery rate < 0.05). All computations were performed using the R statistical software framework (available online: http://www.R-project.org).

### 4.9. Gene Set Enrichment Analysis (GSEA)

GiANT and GSEA were performed as previously reported [22,23,24]. The Gene Expression Omnibus (GEO) dataset GSE121302 representing gene expression analysis from murine liver from *Rbpj^+/+^* and *Rbpj^−/−^* mice were used for the GiANT and the GSEA analysis of the Hippo pathway gene set (mmu04390) and GSE46995 representing gene expression analysis from human BA, non-BA and control patients, was used for the GiANT analysis of the Hippo pathway gene set (hsa04390).

### 4.10. Hepatocyte Isolation and Cultivation

Cells were isolated from adult mouse livers by 2-step collagenase perfusion [55]. Hepatocytes were purified by centrifugation in 50% Percoll (50 g for 10 min at 4 °C). The cell pellet containing the viable cells were washed twice in 20 mL Dulbecco’s modified Eagle medium and centrifuged at 50 g for 5 min at 4 °C. Hepatocytes were cultivated on collagen type I- coated plates in standard Dulbecco’s modified Eagle medium containing 10% fetal bovine serum, 1× Insulin-Transferrin-Selenium X, 10^−7^ mol/L dexamethasone, 1× penicillin/streptomycin/L-glutamine, and 1× nonessential amino acid solution.

### 4.11. Cholestatic Liver Injury In Vitro

500,000 primary hepatocytes from *Rbpj^+/+^* and *Rbpj^−/−^* mice were plated in 6-well plates. 3 h after plating the hepatocytes cells were washed with 1× phosphate-buffered saline (PBS). Immediately we started the treatment with 500 µM GCA for 6 h to induce in vitro cholestatic liver injury. In addition to GCA, cells were also incubated with 10 µM VP to block YAP/TEAD-dependent and 10 µM GSI in inhibit Notch pathway.

### 4.12. Statistical Analysis

The Mann–Whitney test was used to calculate statistical significance using GraphPad Prism 6 (GraphPad Software, Inc, La Jolla, CA, USA) and the data were represented as scatter dot plots (median with interquartile range).

## Supplementary Materials

Supplementary materials can be found at http://www.mdpi.com/1422-0067/19/12/3801/s1.
