# Supplementary material for: YAP Activation Drives Liver Regeneration after Cholestatic Damage Induced by Rbpj Deletion
Source: Int J Mol Sci. 2018 Nov 29;19(12):3801. doi: 10.3390/ijms19123801 (PMC6321044; doi:10.3390/ijms19123801)
Supplement: Supplementary file 1 [file ijms-19-03801-s001.pdf]

Supplementary Figures

Figure S1

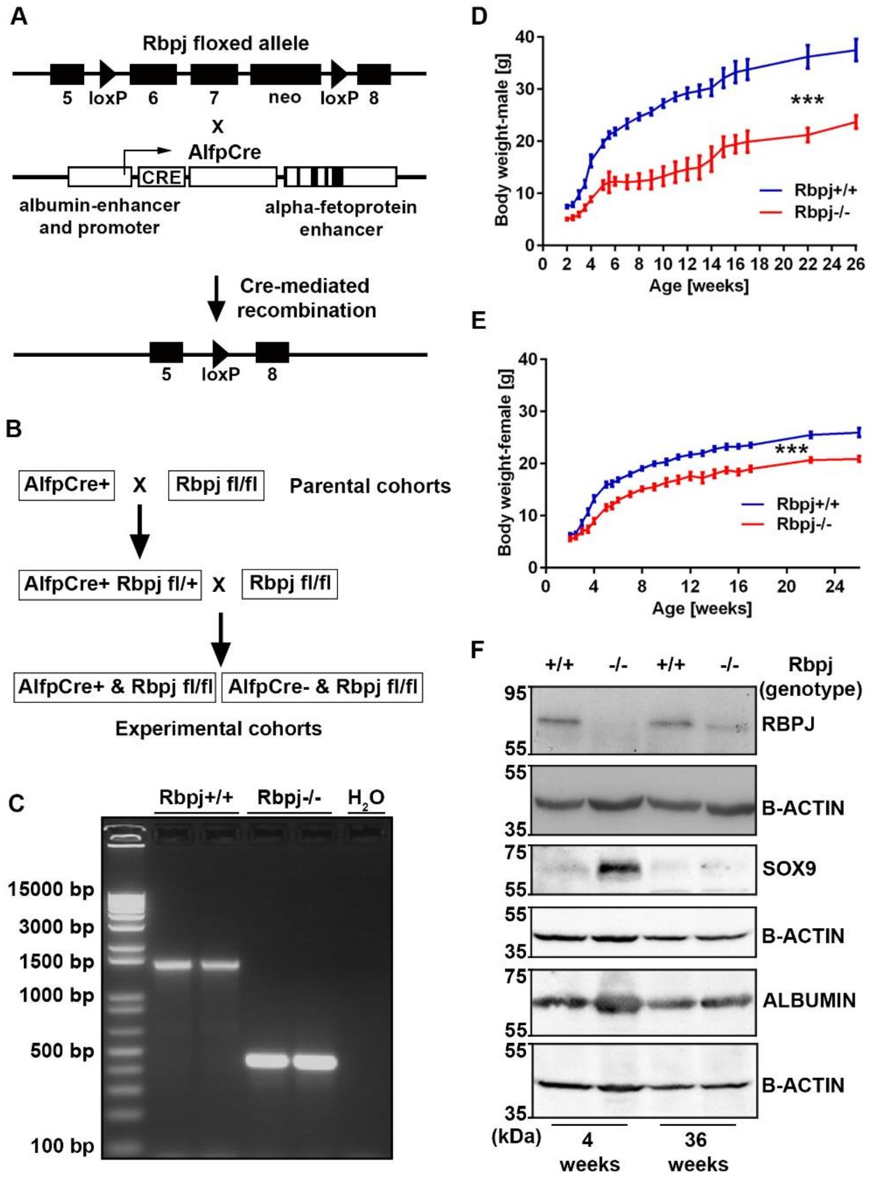

Figure S1. Generation of experimental cohorts and *Rbpj* expression in the liver. (**A**) Scheme of transgenic construct design of conditional *Rbpj* knockout and *AlfpCre* mice [18,19]. (**B**) Breeding strategy for the generation of experimental cohorts. (**C**) PCR analysis of recombination on liver DNA from *Rbpj*$^{+/+}$, and *Rbpj*$^{-/-}$ mice. The *Rbpj* wild-type band is localised at 1500 bp whereas *Rbpj* knockout band can be seen at 490 bp. (**D** + **E**) Weight curves of *Rbpj*$^{+/+}$ and *Rbpj*$^{-/-}$ mice, *Rbpj*$^{-/-}$ mice show a persistent lower body weight in male (n = 9–12; *** $p \leq 0.001$, Pearson coefficient correlation (Two tailed)) (**D**) and female mice (n = 11–16; *** $p \leq 0.001$, Pearson coefficient correlation (Two tailed)) (**E**) group. (**F**) Immunoblot of RBPJ, SOX9 (progenitor/cholangiocytic marker) and Albumin (hepatocytic marker) of whole liver lysates from 4 and 36 weeks old *Rbpj*$^{+/+}$ and *Rbpj*$^{-/-}$ mice (n = 2).

Figure S2

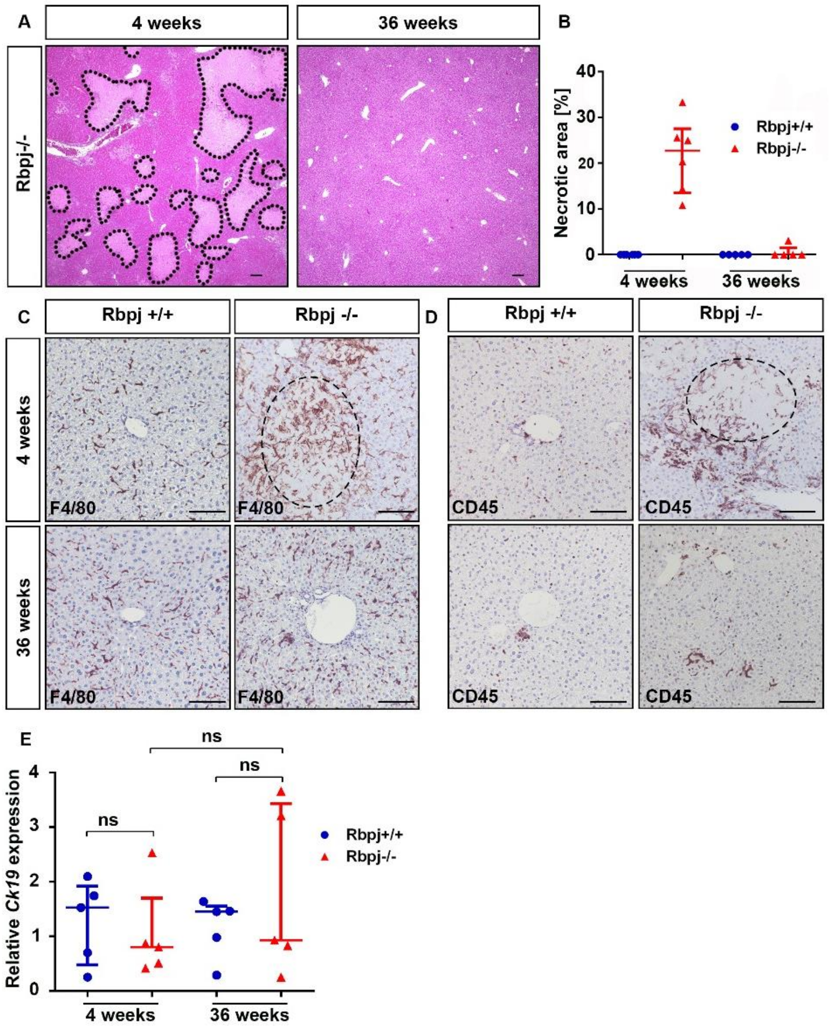

Figure S2. Loss of RBPJ results in hepatic necrosis. (**A**) Photographs of macroscopic liver from *Rbpj*$^{-/-}$ mice at 4 and 36 weeks (n = 5–6; scale bar: 100 µm; dotted lines surround the necrotic areas). (**B**) Scatter dot plots depict the necrotic area in the liver of *Rbpj*$^{-/-}$ mice. *Rbpj*$^{+/+}$ mice did not show necrosis (n = 5–6). (**C**) Representative photographs of F4/80-immunostaining (n = 3; scale bar: 100 µm; dotted line surround the necrotic area). (**D**) CD45-immunostaining of liver from 4 and 36 weeks old *Rbpj*$^{+/+}$ and *Rbpj*$^{-/-}$ mice, Necrotic area is highlighted by dotted line (n = 3; scale bar: 100 µm; dotted lines represent necrotic area). (**E**) Scatter dot plots shows *Ck19* mRNA expression (n = 5; Mann-Whitney test (Two tailed)).

Figure S3

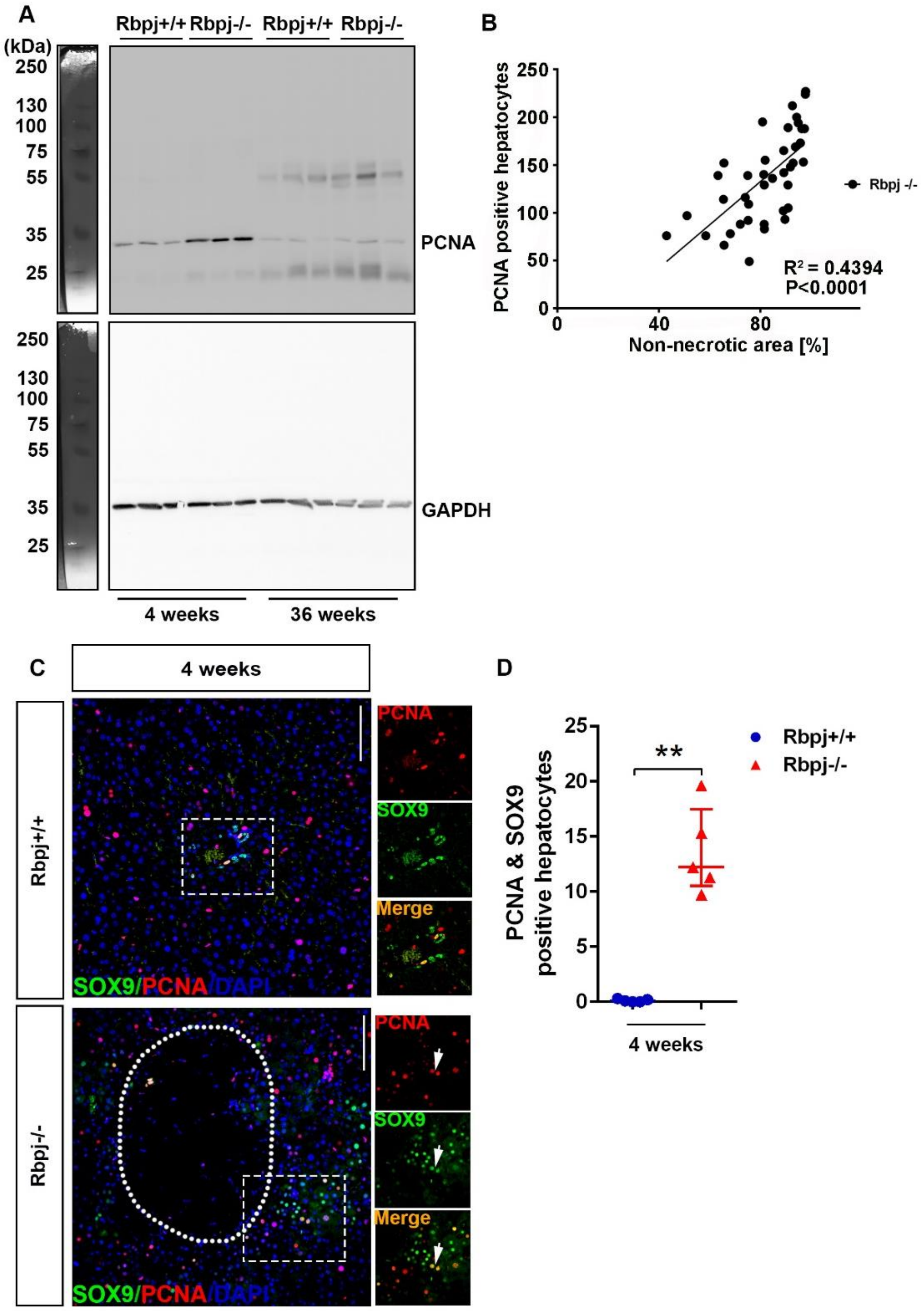

Figure S3. RBPJ deficiency induced cholestasis introduce regenerative proliferation. (**A**) Full-length scan of immunoblot for proliferation marker PCNA of whole liver lysates from *Rbpj*$^{+/+}$ and *Rbpj*$^{-/-}$ mice at 4 and 36 weeks of age. GAPDH was used as a loading control (n = 3). Molecular weight marker is depicted on a separated bright-field image. (**B**) The number of PCNA positive hepatocytes positively correlate with the non-necrotic area in the liver of 4 weeks old *Rbpj*$^{-/-}$ mice (n = 10–11; Pearson correlation coefficient, $R^2 = 0.4394$, $P < 0.0001$). (**C**) Representative photographs of co-immunostaining for the progenitor/cholangiocytic marker SOX9 and the proliferation marker PCNA in liver of 4 weeks old *Rbpj*$^{+/+}$ and *Rbpj*$^{-/-}$ mice (n = 5; scale bar: 100 µm; PCNA: red, SOX9: green, DAPI: blue). The dotted circle represents necrotic area. The marked area is shown as single channels for the detection of PCNA positive cells (red) and SOX9 positive cells (green) and an overlay of both channel. (**D**) Quantification of PCNA and SOX9 positive hepatocytes in liver from 4 weeks old *Rbpj*$^{+/+}$ and *Rbpj*$^{-/-}$ mice (n = 5; ** $p \leq 0.01$, Mann-Whitney test (Two tailed)).

Figure S4

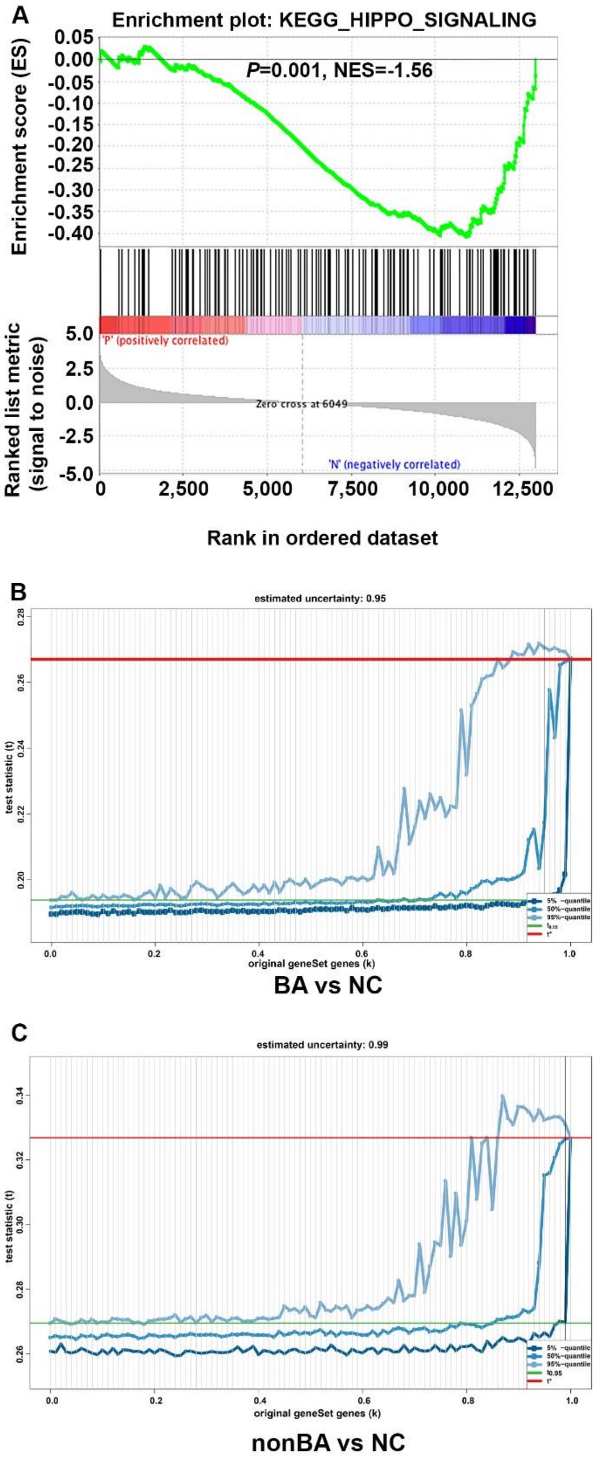

Figure S4. Deregulation of the Hippo pathway gene set in *Rbpj* deleted mice and in human CLD patients. (**A**) Microarray data for liver from 4 weeks old *Rbpj*$^{+/+}$ vs *Rbpj*$^{-/-}$ mice (GSE121302) were analysed using GSEA software to identify significant gene sets. The enrichment plot shows the distribution of genes from the KEGG Hippo pathway gene set (mmu04390). (**B**,**C**) GiANT shows a significant deregulation of the Hippo pathway gene set (hsa04390) in (**B**) BA vs. control patients and (**C**) nonBA vs. control patients.

Figure S5

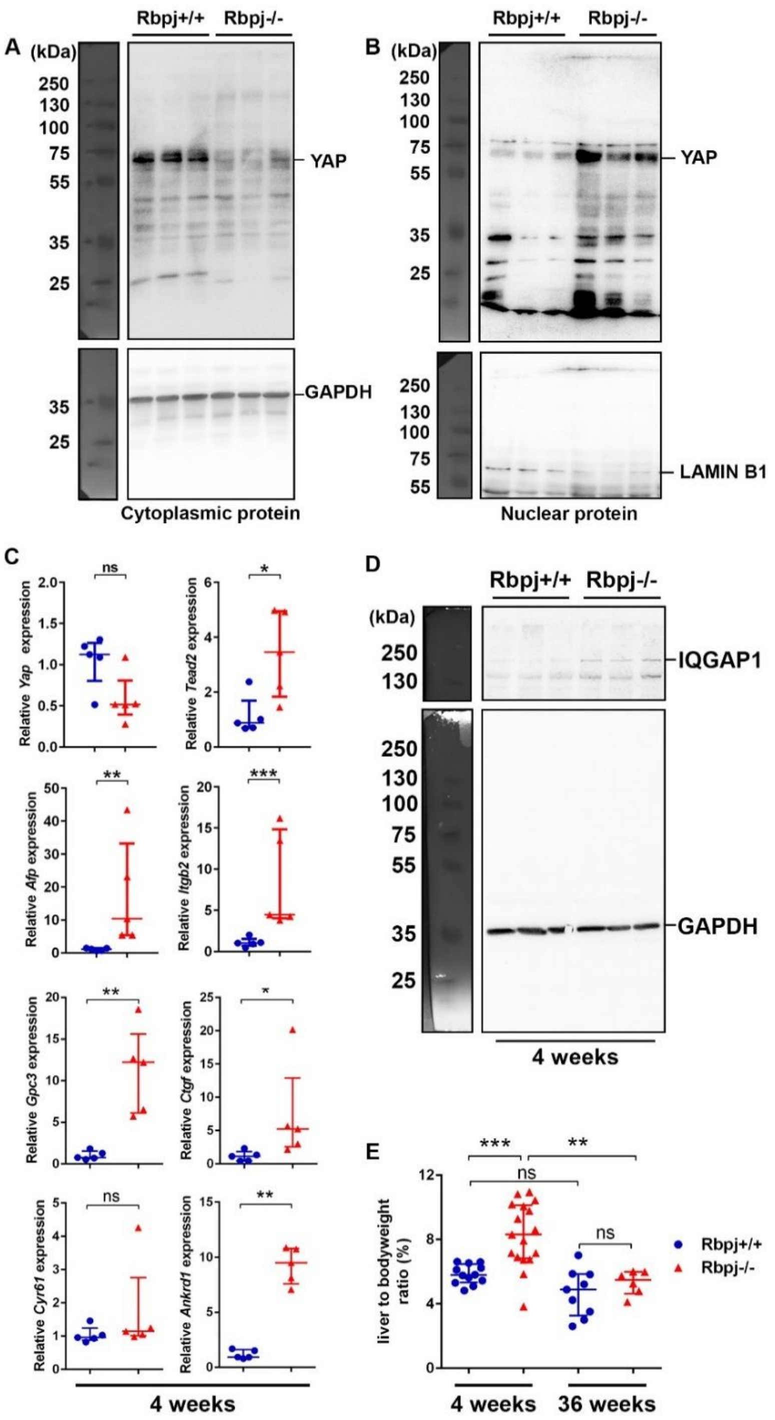

Figure S5. Nuclear translocation of YAP after *Rbpj*$^{-/-}$ induced liver injury. (**A**,**B**) Corresponding full-length scan of immunoblots of cytoplasmic and nuclear YAP in the liver of *Rbpj*$^{+/+}$ and *Rbpj*$^{-/-}$ mice to data presented in cropped Figure 5C. Molecular weight marker is depicted on a separated bright-field image. (**C**) Relative RNA expression of Hippo pathway regulators *Yap* and *Tead2* and YAP downstream targets *Afp*, *Itgb2*, *Gpc3*, *Ctgf*, *Cyr61* and *Ankrd1* (n = 5; *** $p \leq 0.001$, ** $p \leq 0.01$, * $p \leq 0.05$, ns = non-significant; Mann-Whitney test (Two tailed)). (**D**) Full-length scan of immunoblot of IQGAP1 (horizontally cropped) correspond to data presented in cropped Figure 5E. Molecular weight marker is depicted on a separated bright-field image. (**E**) Liver to bodyweight ratio of *Rbpj*$^{+/+}$ and *Rbpj*$^{-/-}$ mice at 4 and 36 weeks of age (n = 6–17; *** $p \leq 0.001$, ** $p \leq 0.01$, ns= non-significant; Mann-Whitney test (Two tailed)).

Figure S6

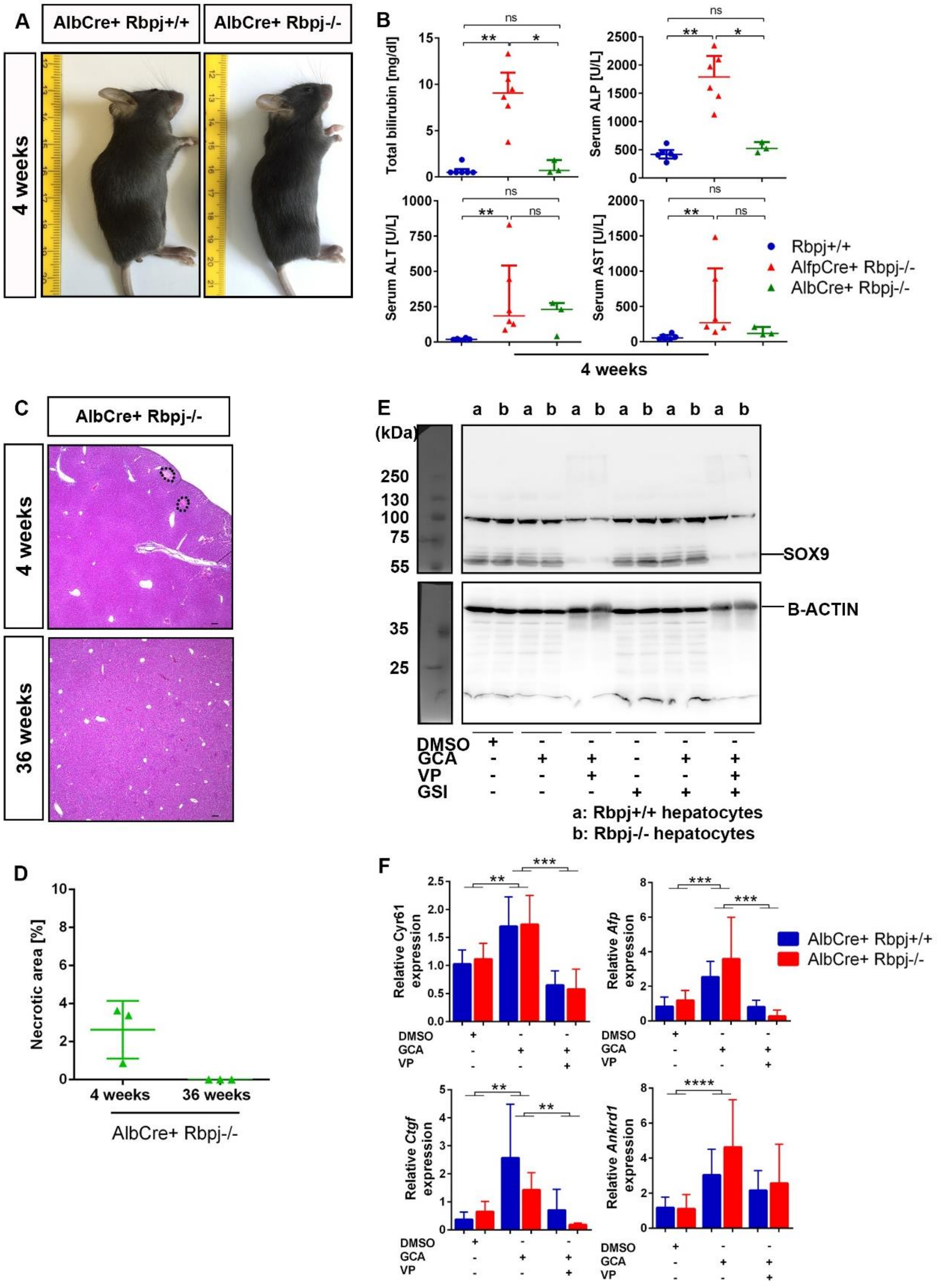

Figure S6. *AlbCre* mediated loss of *Rbpj*. (**A**) Representative macroscopic photographs of *AlbCre*+ *Rbpj*+/+ and *AlbCre*+ *Rbpj*–/– mice at 4 weeks of age (n = 3). (**B**) Scatter dot plots of TB (total bilirubin), ALP (alkaline phosphatase), ALT (alanine transaminase) and AST (aspartate transaminase) measured in mouse serum from 4 weeks old *AlfpCre*+ *Rbpj*–/–, *AlbCre*+ *Rbpj*+/+ and *AlbCre*+ *Rbpj*–/– mice (n = 3-6; ** $p \leq 0.01$, * $p \leq 0.05$, Mann-Whitney test (Two tailed)). Only ALT marker is slightly increased upon *Rbpj* deletion in *AlbCre*+ *Rbpj*–/– mice. (**C**) Representative H&E photographs from *AlbCre*+ *Rbpj*–/– mice at 4 and 36 weeks depicting small necrotic areas in 4 weeks old *AlbCre*+ *Rbpj*–/– mice (n = 3; scale bar: 100 μm, dotted lines represents necrotic areas). (**D**) Scatter dot plots represent the necrotic area in the liver

of *AlbCre*+ *Rbpj*−/− mice (n = 3). (**E**) Corresponding full-length scan of immunoblot is horizontally cropped for the separate detection of SOX9 and β-Actin (whole-cell protein). Correspond to data presented in cropped Figure 6D. Molecular weight marker is depicted on a separated bright-field image. (**F**) Relative RNA expression of *Cyr61*, *Afp*, *Ctgf* and *Ankrd1* in primary hepatocytes from *AlbCre*+ *Rbpj*+/+ and *AlbCre*+ *Rbpj*−/− mice after GCA and GCA/VP treatment (n = 5; **** $p \leq 0.0001$, *** $p \leq 0.001$, ** $p \leq 0.01$, * $p \leq 0.05$, Mann-Whitney test (Two tailed)).

Supplementary Tables

Table S1. Enriched KEGG pathways.

KEGG-Pathways	Gene Size	Intersection Size	False Discovery Rate
METABOLIC_PATHWAYS	1230	101	4.40E-10
PI3K-AKT_SIGNALING_PATHWAY	355	37	2.27E-06
PATHWAYS_IN_CANCER	326	29	0.000602417
HTLV-I_INFECTION	290	28	0.000142525
CYTOKINE-CYTOKINE_RECEPTOR_INTERACTION	273	31	1.23E-06
MAPK_SIGNALING_PATHWAY	259	25	0.000497632
VIRAL_CARCINOGENESIS	236	16	0.04207037
ENDOCYTOSIS	225	17	0.026686827
EPSTEIN-BARR_VIRUS_INFECTION	220	17	0.018968375
REGULATION_OF_ACTIN_CYTOSKELETON	217	26	1.44E-05
FOCAL_ADHESION	205	33	1.38E-09
ALCOHOLISM	202	16	0.005436578
CHEMOKINE_SIGNALING_PATHWAY	198	29	1.46E-08
CALCIUM_SIGNALING_PATHWAY	185	15	0.02930663
TRANSCRIPTIONAL_MISREGULATION_IN_CANCER	181	16	0.007818261
PHAGOSOME	180	31	4.40E-10
TUBERCULOSIS	179	25	1.57E-06
INFLUENZA_A	172	15	0.013058534
HIPPO_SIGNALING_PATHWAY	156	14	0.017665702
NATURAL_KILLER_CELL_MEDIATED_CYTOTOXICITY	155	20	2.43E-05
HEPATITIS_B	149	17	0.000662977
SYSTEMIC_LUPUS_ERYTHEMATOSUS	147	17	4.02E-05
INSULIN_SIGNALING_PATHWAY	140	12	0.033808161
VASCULAR_SMOOTH_MUSCLE_CONTRACTION	136	12	0.029256413
AXON_GUIDANCE	133	14	0.005272863
SEROTONERGIC_SYNAPSE	130	13	0.007977282
CELL_CYCLE	129	21	1.00E-06
OSTEOCLAST_DIFFERENTIATION	127	25	1.75E-09
LYSOSOME	124	20	2.26E-06
NEUROTROPHIN_SIGNALING_PATHWAY	123	13	0.006348471
LEUKOCYTE_TRANSENDOTHELIAL_MIGRATION	121	21	6.00E-07
AMOEBIASIS	120	26	4.40E-10
OOCYTE_MEIOSIS	115	13	0.003392808
CHOLINERGIC_SYNAPSE	114	10	0.043511615
HIF-1_SIGNALING_PATHWAY	113	15	0.000289593
TOXOPLASMOSIS	113	11	0.021591468

T_CELL_RECEPTOR_SIGNALING_PATHWAY	110	13	0.002701362
CHAGAS_DISEASE	104	15	0.000199555
NF-KAPPA_B_SIGNALING_PATHWAY	103	12	0.001673957
TOLL-LIKE_RECEPTOR_SIGNALING_PATHWAY	101	12	0.003467801
CIRCADIAN_ENTRAINMENT	99	10	0.021591468
ARACHIDONIC_ACID_METABOLISM	98	14	0.000216025
DRUG_METABOLISM_CYTOCHROME_P450	97	16	5.67E-06
METABOLISM_OF_XENOBIOTICS_BY_CYTOCHROME_P450	96	19	6.61E-08
CHEMICAL_CARCINOGENESIS	95	17	1.23E-06
FC_GAMMA_R-MEDIATED_PHAGOCYTOSIS	92	22	9.77E-10
PROGESTERONE	89	9	0.029078089
GNRH_SIGNALING_PATHWAY	89	9	0.029673656
PROSTATE_CANCER	89	9	0.029673656
GAP_JUNCTION	88	13	0.000359935
PROTEIN_DIGESTION_AND_ABSORPTION	88	10	0.011079573
ECM-RECEPTOR_INTERACTION	87	15	3.41E-05
HEMATOPOIETIC_CELL_LINEAGE	87	11	0.00336888
ERBB_SIGNALING_PATHWAY	87	11	0.003790869
SMALL_CELL_LUNG_CANCER	86	11	0.003488665
RHEUMATOID_ARTHRITIS	84	13	0.000257738
RETINOL_METABOLISM	83	11	0.000978177
TGF-BETA_SIGNALING_PATHWAY	83	8	0.048932522
PPAR_SIGNALING_PATHWAY	81	11	0.002127223
PEROXISOME	81	9	0.017665702
PHOSPHATIDYLINOSITOL_SIGNALING_SYSTEM	81	9	0.020136032
SALMONELLA_INFECTION	79	11	0.001937438
B_CELL_RECEPTOR_SIGNALING_PATHWAY	78	13	0.000114631
SALIVARY_SECRETION	77	10	0.005335128
COMPLEMENT_AND_COAGULATION_CASCADES	76	14	2.12E-05
ADHERENS_JUNCTION	75	9	0.010951802
CHRONIC_MYELOID_LEUKEMIA	74	9	0.010217549
PERTUSSIS	73	13	7.13E-05
MELANOMA	73	9	0.008628681
BILE_SECRETION	72	11	0.001064948
FC_EPSILON_RI_SIGNALING_PATHWAY	71	11	0.000786329
BACTERIAL_INVASION_OF_EPITHELIAL_CELLS	70	9	0.007977282
P53_SIGNALING_PATHWAY	69	7	0.046484703
RENAL_CELL_CARCINOMA	69	7	0.046484703
PANCREATIC_CANCER	67	12	0.000139531
LEISHMANIASIS	66	15	1.23E-06
GLIOMA	66	12	0.00011098
GLYCOLYSIS_GLUCONEOGENESIS	66	10	0.001406944
VEGF_SIGNALING_PATHWAY	66	7	0.044228701
COLORECTAL_CANCER	64	10	0.00157458
DRUG_METABOLISM_OTHER_ENZYMES	61	8	0.005312502
INOSITOL_PHOSPHATE_METABOLISM	61	7	0.033808161
LEGIONELLOSIS	59	9	0.002620862
NOD-LIKE_RECEPTOR_SIGNALING_PATHWAY	59	7	0.028680537
STEROID_HORMONE_BIOSYNTHESIS	58	14	3.52E-07
GLYCEROLIPID_METABOLISM	56	8	0.006348471

MALARIA	55	11	8.15E-05
GLUTATHIONE_METABOLISM	55	10	0.000410474
NON-SMALL_CELL_LUNG_CANCER	55	8	0.005771628
STARCH_AND_SUCROSE_METABOLISM	53	10	0.000114631
STAPHYLOCOCCUS_AUREUS_INFECTION	52	14	2.92E-07
FANCONI_ANEMIA_PATHWAY	52	6	0.042288568
ENDOMETRIAL_CANCER	52	6	0.045035893
TYPE_II_DIABETES_MELLITUS	50	7	0.014378328
AMINO_SUGAR_AND_NUCLEOTIDE_SUGAR_METABOLISM	49	7	0.010596964
FATTY_ACID_METABOLISM	48	7	0.00959166
ABC_TRANSPORTERS	46	6	0.030660707
PORPHYRIN_AND_CHLOROPHYLL_METABOLISM	43	8	0.000617247
PYRUVATE_METABOLISM	43	7	0.006880665
SPHINGOLIPID_METABOLISM	41	8	0.001358947
FRUCTOSE_AND_MANNOSE_METABOLISM	40	8	0.001002815
ALDOSTERONE-REGULATED_SODIUM_REABSORPTION	40	7	0.004986944
BASE_EXCISION_REPAIR	39	5	0.029673656
DNA_REPLICATION	36	9	9.60E-05
PRION_DISEASES	35	7	0.002506143
PENTOSE_AND_GLUCURONATE_INTERCONVERSIONS	34	12	1.51E-08
GALACTOSE_METABOLISM	31	7	0.00129682
BUTANOATE_METABOLISM	31	5	0.021647451
ASCORBATE_AND_ALDARATE_METABOLISM	27	6	0.000978177
VITAMIN_DIGESTION_AND_ABSORPTION	24	4	0.040651071
MISMATCH_REPAIR	23	4	0.031060801
GLYCOSAMINOGLYCAN_DEGRADATION	21	4	0.028680537
SULFUR_METABOLISM	11	4	0.003418509
SYNTHESIS_AND_DEGRADATION_OF_KETONE_BODIES	11	3	0.020651069

Table represents list of enriched KEGG pathways in whole liver tissue of *Rbpj*$^{+/+}$ and *Rbpj*$^{-/-}$ mice at the age of 4 weeks. Table shows gene set size, intersection size and false discovery rate for each single pathway (n = 3–4, Fisher's exact test, fdr < 0.05).

Table S2. Differentially regulated genes.

Gene	Log2 Fold Change	False Discovery Rate
SULT2A1	9.851442969	3.06E-09
SULT2A4	9.808841664	1.35E-13
SULT2A2	9.700201106	3.39E-08
ENSMUST00000121135	8.126654798	1.35E-13
LRTM2	7.150394141	2.44E-09
IGDCC4	7.011721889	2.44E-09
H19	6.803596774	0.000954713
GIPC2	6.71165252	5.44E-06
KRT20	6.526805469	1.35E-13
SPINK3	6.512761648	1.35E-13
SULT1E1	6.453821301	0.028990591

SULT2A6	6.168625668	6.90E-10
SCD2	5.954048258	1.21E-11
LCN2	5.641675788	0.005818331
TCEAL5	5.559467642	5.44E-07
UBD	5.548400939	0.024306258
HKDC1	5.302800512	1.85E-06
S100G	5.265316127	1.31E-06
BMPER	5.141137394	0.005818331
TIMP1	5.135291809	0.099516918
ENSMUST00000040703	4.966763664	1.35E-13
AKR1B7	4.921120317	0.005818331
ELOVL7	4.885913221	1.32E-07
GPNMB	4.863316341	0.003229661
GLOD5	4.850266376	1.35E-13
GM3776	4.704583134	0.000774517
OSBPL3	4.69758438	1.35E-13
PRR15L	4.618857373	1.35E-13
5730559C18RIK	4.618355655	6.75E-06
SLC22A29	4.611978031	0.014937438
CD14	4.589637345	0.00216544
ORM3	4.547618187	7.86E-08
MYOM3	4.46650509	0.001466487
ELF3	4.438428827	0.009923494
CCL2	4.435052061	0.003229661
IFNLR1	4.425924904	1.35E-13
SPRR1A	4.418043304	0.001466487
RASAL1	4.367456938	3.06E-09
BEX1	4.310830819	0.001466487
PPL	4.307147269	1.35E-13
ARHGAP22	4.163173582	2.44E-09
PBK	4.161804206	0.007581085
CHIL1	4.160336118	3.84E-05
IL13RA2	4.136030848	8.29E-11
WNT10A	4.110943424	1.35E-13
PYGB	4.089629132	7.10E-05
BC100530	4.08803171	5.69E-06
CXCL1	3.992729169	0.0734861
CXCL5	3.964574092	1.35E-13
IFI27L2B	3.934879342	1.35E-13
TOP2A	3.896093594	0.005818331
CIDEA	3.895105822	0.002188424
PAK1	3.886654951	0.005147085
SLC51B	3.885615298	1.77E-07
ENSMUST00000095071	3.863177767	0.099516918
FFAR4	3.837122129	0.000346571
TUBB3	3.828369714	0.000226234
STFA2L1	3.828120579	0.014937438
CALML4	3.81756269	1.35E-13
TNFAIP3	3.813479821	0.005818331

GM10639	3.785127557	0.007581085
SMPD3	3.775204622	1.21E-11
MYO7B	3.749274494	4.87E-10
FOS	3.737032347	5.44E-07
4930415O20RIK	3.724876421	0.014937438
RNF138RT1	3.709451094	0.000346571
CCDC120	3.70443112	0.005818331
A_55_P2024391	3.6983378	0.037937888
ENSMUST00000077110	3.691780728	1.40E-06
RETNLG	3.688870132	0.005818331
ADAMTS4	3.66547627	1.35E-13
GPX3	3.638837874	0.000167342
CACNA2D4	3.629506089	1.35E-13
AUNIP	3.619373802	0.000774517
SH3PXD2B	3.610954145	0.005818331
EMB	3.600907112	0.004840579
TREM2	3.584377671	0.007581085
EPDR1	3.571531964	0.000346571
PDK3	3.57061184	0.000226234
SERPINB6D	3.568395122	1.71E-05
TC1661613	3.563238318	0.099516918
TFF3	3.562779006	1.35E-13
MGST2	3.528877289	0.007581085
NEDD9	3.527107015	0.001466487
CX3CR1	3.525954373	0.007581085
4930427A07RIK	3.517972255	0.005818331
PLAUR	3.508837964	0.029429781
STFA1	3.504006539	0.023459349
EXO1	3.499500448	0.003669217
1700016C15RIK	3.465034151	6.15E-07
CD36	3.462142373	1.35E-13
GM5483	3.452850276	7.10E-05
INPP5J	3.448146668	0.004640056
GSTA2	3.445770597	0.053870316
RAD51	3.445443049	0.099516918
FIGNL1	3.425772797	0.000506785
IER3	3.421857578	0.0734861
EPS8L3	3.403922179	4.86E-05
ADAMTS15	3.403736278	0.003669217
IRAK3	3.363356686	0.0734861
9130017K11RIK	3.355272467	3.24E-07
WFDC3	3.349986727	0.037937888
SERPINB6B	3.349939683	0.014937438
GRRP1	3.346006158	1.37E-05
SERPINB6A	3.339463191	1.35E-13
IL1R2	3.330651384	6.75E-05
SERPINB6C	3.324530061	3.39E-08
NRG1	3.307193354	0.002188424
CCL7	3.294318303	0.028990591

BUB1	3.28491747	0.004640056
RAB44	3.277317802	0.000774517
FERMT3	3.238495676	0.0734861
CCL6	3.236960306	0.000166554
9530077C05RIK	3.179569445	1.35E-13
NTF3	3.172695952	0.024306258
IQGAP3	3.161702649	0.002188424
ALPK1	3.157341205	5.44E-07
ANLN	3.152704056	0.000954713
ENSMUST00000111315	3.151923355	5.44E-06
MARCO	3.151582738	0.000954713
ATF3	3.132022346	0.001466487
WFDC16	3.130901213	0.037937888
BUB1B	3.100235562	0.005818331
PLXNA3	3.099028764	0.002188424
PI16	3.096679485	6.15E-07
CHR11:35805295-35817263_F	3.089096117	1.77E-07
SCUBE1	3.076851287	0.001324416
SERPINA7	3.073277823	0.024803337
BMP8B	3.071683723	0.011102126
CTSJ	3.071457146	0.002188424
TREH	3.071412579	0.005818331
ADRB2	3.047740544	0.014937438
CNN1	3.047632532	0.000954713
SMOC2	3.044138357	1.35E-13
EID2	3.033634113	0.024803337
C1QTNF6	3.02524307	3.06E-09
FGL2	3.024342084	0.007581085
CD300LB	3.012551457	6.90E-10
FAM105A	3.010758303	1.32E-07
SIRPB1B	3.010224915	0.000954713
ABCB1A	2.998166661	6.15E-07
GUCA2B	2.994088808	0.043182203
DTL	2.993434022	0.099516918
AA986860	2.980665993	0.005818331
PRC1	2.977956398	5.44E-06
LYSMD2	2.96229441	0.099516918
ENSMUST00000043503	2.96193716	0.079872805
ENSMUST00000143791	2.960076437	1.40E-06
CLEC4D	2.951017283	1.40E-06
GOLM1	2.940913134	1.35E-13
PLK1	2.937737032	0.000774517
AFP	2.934659184	6.15E-07
1810053B23RIK	2.932434462	0.000954713
B4GALT6	2.931551654	0.000774517
RASD1	2.927395989	0.014937438
SDCBP2	2.923038712	0.002188424
PLA2G7	2.920718163	7.02E-09
NEURL3	2.919983687	0.011102126

MSANTD3	2.917275586	1.32E-07
ENSMUST00000107229	2.910285695	0.0734861
ERCC6L	2.907878012	1.77E-07
LYZ2	2.903452675	5.44E-06
TAGLN2	2.901081208	1.21E-11
POLQ	2.897567431	0.003669217
SGOL2A	2.897412856	0.014937438
CENPI	2.894453814	0.014937438
POPDC2	2.879367817	0.005818331
RAB31	2.873682052	0.004640056
ENSMUST00000181735	2.870095284	0.000162979
B3GALNT1	2.868151103	0.000506785
DEPDC1B	2.867999979	0.0734861
NEU3	2.863996239	0.014937438
EGR2	2.863440041	0.043182203
MFGE8	2.854371257	1.35E-13
MXD3	2.849724371	0.000346571
IFITM6	2.849310661	0.000346571
TNFRSF12A	2.839123601	0.014937438
TBX1	2.827572756	1.35E-13
RCAN2	2.827561302	5.44E-06
2210011C24RIK	2.818866562	7.10E-05
ANXA2	2.818686615	4.87E-10
A_55_P2034227	2.81255734	3.24E-07
PLS1	2.807355522	0.000162979
CCDC3	2.800977843	3.84E-05
MCM5	2.793541996	0.0734861
BICC1	2.782471656	4.85E-12
SIRPB1A	2.773756482	0.005147085
AURKA	2.77329198	1.37E-05
ENSMUST00000120059	2.764008522	0.000346571
TMEM139	2.758032341	0.002188424
NOXA1	2.75192591	5.44E-07
FANCI	2.745316041	0.029429781
UBE2C	2.744294268	0.002188424
CCR2	2.731082813	0.024306258
S100A11	2.729704194	6.76E-06
ESPL1	2.724320454	0.059268219
CLCA3A2	2.713650725	3.06E-09
RGS1	2.713485002	0.002188424
DDR1	2.713303732	0.029429781
ADGRG1	2.712092325	1.32E-07
NUSAP1	2.70790881	0.029429781
BCL2A1C	2.69745698	0.014937438
C5AR1	2.692966874	0.037937888
SERPINA3I	2.692314701	0.011102126
PMAIP1	2.689201987	0.0734861
TFF2	2.687357642	0.001466487
SLC41A3	2.675868467	6.75E-05

NCAPG2	2.672822174	0.011102126
CYB561	2.664675116	0.000167342
VILL	2.663615743	0.000162979
PLP2	2.658259741	0.079872805
LGALS3	2.658213121	0.000189142
NAP112112-1	2.654966056	0.002188424
SLC16A3	2.651489674	0.000954713
KRT31	2.650668509	0.000346571
OLIG1	2.648731722	0.005818331
NMUR2	2.648079969	0.000107205
ENSMUST00000190026	2.642635882	1.71E-05
CLEC4E	2.638392671	3.39E-08
GPRC5B	2.630784972	0.000162979
CEBPD	2.615604802	0.059268219
NID1	2.611497703	4.86E-05
LRR1	2.60999886	0.005818331
CHR5:35893909-35899497_F	2.599027408	0.014937438
TBC1D9	2.598901281	6.75E-05
PKIA	2.585466809	0.002188424
MUC13	2.580743259	0.000506785
THBD	2.573627626	0.059268219
CD63	2.573422875	0.005818331
TNFRSF21	2.569137888	0.0734861
ASPRV1	2.569073329	1.37E-05
MS4A7	2.567315661	1.37E-05
CSF2RB2	2.566636589	0.009923494
ALS2CR12	2.562546422	0.079872805
GALNT3	2.558859486	0.000107205
CD53	2.5510666	0.0734861
DSCC1	2.548155987	0.024306258
A_55_P2050044	2.545196062	0.059268219
CCNA2	2.540735498	0.005818331
P3H2	2.537922227	0.000954713
SDK1	2.523414177	1.35E-13
CLEC1A	2.518529853	0.002188424
ANXA5	2.512818558	3.06E-09
CHR9:78104935-78116974_F	2.506685355	0.0734861
MCM2	2.499787054	0.002188424
PROM1	2.498473561	0.0734861
ACNAT2	2.4934596	0.000954713
ENTPD1	2.493326391	1.35E-13
ATP11A	2.485947602	0.003229661
CENPH	2.485767617	0.043182203
NPDC1	2.483546971	1.45E-11
KNTC1	2.477569049	0.037937888
SYT12	2.47267556	7.99E-11
RAB3B	2.47227438	0.003229661
COL4A3	2.470712509	0.024306258
A_55_P2063146	2.454585957	0.005818331

B930041F14RIK	2.443567229	0.003669217
MS4A8A	2.441472037	8.29E-11
CLIC6	2.438500968	1.35E-13
MTMR11	2.437003404	0.000107205
ISYNA1	2.434148608	5.44E-07
MKI67	2.433121319	0.001466487
3110035E14RIK	2.424095173	0.089012317
ACMSD	2.423451052	0.014937438
5330417C22RIK	2.420952158	3.73E-05
SHCBP1	2.418690505	0.028990591
TMEM98	2.413279075	8.29E-11
FAM64A	2.404403079	0.024306258
MMP8	2.400737006	0.028990591
CRYM	2.393590916	0.007581085
ASPM	2.387600083	0.099516918
GAL3ST1	2.387086549	0.004640056
ATP6V0D2	2.379044725	0.099516918
GJA1	2.377121679	0.024306258
NAPSA	2.375906721	0.037937888
CDCA5	2.373872285	8.57E-06
CBR2	2.369802092	0.012257839
CLDN7	2.368872909	0.043182203
PODXL	2.368473649	1.35E-13
TMPRSS2	2.368035131	0.000346571
ENSMUST00000193767	2.364894584	0.001466487
TFRC	2.360355015	0.028990591
GM11937	2.358787431	2.81E-09
GRAMD1B	2.350771308	1.71E-05
RAB39	2.349016671	0.029429781
ANKRD1	2.344369396	0.099516918
BEX4	2.341041405	0.000346571
OSMR	2.340684757	0.000506785
CKAP2	2.338714036	0.079872805
RACGAP1	2.336785544	0.011102126
IGFBP1	2.336540455	1.37E-05
A_55_P2025829	2.332756421	1.37E-05
ENC1	2.332277722	0.002188424
A_55_P2038452	2.331117163	0.043182203
MS4A6D	2.326695458	0.005818331
STMN1	2.32263545	0.029429781
CCDC80	2.320798009	0.005147085
RDH9	2.319065571	6.15E-07
CLDN6	2.316278783	0.004840579
ZC2HC1A	2.314089436	0.000189142
IL33	2.313432401	0.079872805
ACOT10	2.311647445	0.005147085
CENPE	2.311118626	0.003229661
ALOX5	2.304598833	1.40E-06
BCL2A1D	2.292360739	6.15E-07

PANX1	2.286362694	1.71E-05
FCGR3	2.283678418	3.24E-07
CBR3	2.282174876	0.024306258
A_55_P2020072	2.270358587	0.003669217
GATSL3	2.268481458	0.000107205
SGOL1	2.266324266	0.000346571
FHL2	2.261117427	0.037937888
PARPBP	2.258839977	0.029429781
ADAM8	2.25243597	0.009923494
P2RY14	2.249965227	0.009923494
LPL	2.24877024	3.39E-08
MCM4	2.24809439	0.003229661
HAVCR2	2.24735062	0.099516918
ENSMUST00000165104	2.242351332	8.57E-06
EXOC3L4	2.241223799	0.0734861
CAPZA2	2.239066977	0.007581085
A_55_P2005972	2.219323359	0.029429781
ENSMUST00000191760	2.219113317	6.15E-07
TM4SF4	2.189096747	1.45E-11
CENPT	2.188763191	0.002188424
RND2	2.185049013	0.005818331
GSTA4	2.180937039	0.024306258
CYTIP	2.177807764	0.005147085
CD93	2.174159011	0.003229661
NAP111417-1	2.17009075	6.76E-06
FADS3	2.160571563	0.000346571
5730416F02RIK	2.155339292	3.24E-07
WFDC17	2.150949611	0.000506785
GM5936	2.149201439	0.000189142
ENSMUST00000022099	2.148754164	0.099516918
OIP5	2.147393432	0.049978137
DXBAY18	2.146888686	0.005147085
GM9733	2.143407529	0.001324416
TACC3	2.142451997	0.000774517
SERPINA3N	2.139315271	0.023459349
SHC2	2.138790023	0.0734861
CDC25C	2.135831692	0.0734861
THBS2	2.135435512	0.028990591
CTTNBP2NL	2.134806773	0.087334443
ABCC1	2.132272953	2.81E-09
UAP1L1	2.13002399	0.005147085
IL1B	2.123385804	0.000107205
CLEC4N	2.119078908	5.44E-07
ENSMUST00000118082	2.116850568	0.000954713
A_55_P2145136	2.112251924	0.000506785
NR4A1	2.107821576	0.014937438
ARHGEF39	2.107246367	0.0734861
TPPP3	2.096904173	0.024306258
MCM6	2.096614889	0.000107205

PPAP2A	2.095120604	6.76E-06
TROAP	2.093365294	0.099516918
ABCC4	2.090107183	1.71E-05
ADM	2.090073929	0.000107205
CD5L	2.088859896	8.57E-06
GLIPR1	2.086201245	0.014937438
KIF22	2.082529784	0.005818331
EVPL	2.08179842	0.0734861
MYO5A	2.077041575	0.099516918
GM5150	2.076481668	0.024306258
CAR13	2.076289945	6.85E-07
CHR2:77715502-77719430_F	2.075473423	0.014937438
LOC102642410	2.068425986	0.043182203
CFAP69	2.068316691	1.10E-09
TMEM106A	2.066502028	0.003669217
DIAP3	2.062364689	0.005818331
LUM	2.060916111	0.000346571
PIK3R5	2.058507302	0.015085602
EMILIN2	2.057501519	0.001538172
CLEC4A3	2.05373857	0.0734861
A_55_P2002460	2.048723989	0.028990591
SCN1B	2.04803695	0.005818331
GM38958	2.047386057	5.44E-06
EREG	2.047092812	0.005818331
CLEC4B1	2.044436314	0.024803337
CTSS	2.041781943	3.39E-08
SMC2	2.038614929	7.10E-05
FABP7	2.036288825	7.02E-09
SLC44A4	2.035156757	0.043182203
CD83	2.032356422	0.012257839
ASF1B	2.030872261	3.06E-09
RAPGEF5	2.029560838	0.001466487
H2-Q1	2.029293822	3.73E-05
VWF	2.028021189	0.000166554
SGK1	2.023620789	0.014937438
HN1	2.022928076	0.002188424
VCAM1	2.018648813	0.029429781
ACTG2	2.015810408	0.023459349
SPON1	2.014496794	0.005147085
RNASEL	2.011058417	0.099516918
C1QB	2.009530539	1.31E-06
ORM2	2.005426831	0.005818331
CD38	2.005065845	3.39E-08
CHR19:61226425-61270975_F	2.001785373	0.0734861
NCAPH	2.00132479	1.77E-07
TYROBP	1.999491895	3.39E-08
GM11223	1.998944114	0.037937888
LILRB4	1.997389095	0.005147085
DAPP1	1.99682631	1.20E-06

PLET1	1.993582474	0.003229661
LY6E	1.991561674	8.29E-11
CNR1	1.991351882	0.037937888
LGALS1	1.987735615	4.87E-10
LRRC75A	1.98706649	0.014937438
AMZ1	1.985401854	0.000506785
TLR2	1.980283335	0.001466487
EAR1	1.979334002	1.85E-06
VAV1	1.977964049	0.003669217
ACTG1	1.972301746	0.000167342
PLAT	1.971790864	0.037937888
KIF20B	1.969997506	0.023459349
GPR176	1.966939826	1.60E-10
GINS2	1.966843274	0.0734861
ADGRE1	1.965079045	5.44E-06
PPM1J	1.959156135	0.005818331
LACC1	1.959032012	0.000506785
H2-DMA	1.957824052	0.002188424
REEP1	1.956940487	0.029429781
LYZ1	1.95568612	0.004840579
CFAP45	1.953883665	0.099516918
KRT8	1.952221767	0.000107205
SPEER4B	1.950069564	1.37E-05
NIPA1	1.94899761	0.029429781
RGS19	1.947961735	0.000954713
CYBB	1.945144731	0.005818331
KLF6	1.940435365	0.005147085
A_55_P2063426	1.939661746	0.002188424
EME1	1.930259202	0.059268219
PLAC8	1.929252357	8.57E-06
CDC6	1.921630542	0.028990591
RELB	1.919791565	7.10E-05
TFEC	1.917058298	0.043182203
CAND2	1.916537594	0.014937438
ABCG1	1.915119305	1.37E-05
CHRDL2	1.914483602	0.007581085
ACOT9	1.910758996	0.000774517
4632428N05RIK	1.907776431	0.005147085
ALDH1B1	1.905290036	0.009923494
A_55_P1970596	1.905197662	1.37E-05
SLC15A3	1.904836013	6.15E-07
ENSMUST00000033486	1.904129822	0.001466487
RRM2	1.903341585	0.002188424
VAV3	1.901773492	0.053870316
INCENP	1.901683535	1.32E-07
GALNT12	1.899873682	0.003229661
CHR9:78107225-78118850_F	1.897897278	0.003229661
STK10	1.89178193	3.06E-09
ADGRL4	1.890925468	0.002188424

JDP2	1.88122218	0.003669217
TRPM6	1.877572893	0.0734861
NCAPD2	1.877566278	3.84E-05
BTBD11	1.876669551	2.44E-09
MYO1F	1.876537946	0.000166554
ARG2	1.86885278	0.043182203
GM13272	1.868813562	0.0734861
EFCAB9	1.867676125	4.87E-10
TMEM246	1.86541251	0.007581085
ASB11	1.864669375	0.0734861
EHD4	1.861157025	0.024306258
LMO1	1.86108632	0.087334443
GSTM3	1.859017653	5.44E-06
IL18BP	1.85812884	1.77E-07
CCND1	1.857258085	0.005147085
RCAN1	1.854409841	6.76E-06
CYBA	1.847114476	1.37E-05
ZWILCH	1.846855544	0.023459349
CDCA8	1.844440848	0.024306258
IGF2BP3	1.840775469	0.023459349
NAIP2	1.837697657	2.44E-09
OBOX8	1.835239957	0.0734861
NLN	1.833373212	0.099516918
SLC5A6	1.828386046	0.007581085
RHOC	1.827707742	0.000107205
CERK	1.82757736	6.75E-05
MGST3	1.827259513	0.002188424
CYP4F18	1.824765137	0.000954713
SLC11A1	1.823751122	6.75E-06
HEXIM1	1.822242875	5.69E-06
NCF1	1.820134606	0.014937438
KIFC5B	1.819698284	0.029429781
E230029C05RIK	1.807468382	0.001466487
HMGB2	1.807466149	1.20E-06
TMEM240	1.806716801	0.024306258
MCM3	1.804945179	0.005818331
DMPK	1.802340473	6.15E-07
STK17B	1.796780371	0.000954713
CHR3:146645022-146646977_F	1.796129052	0.099516918
ID4	1.79610855	0.0734861
GM9292	1.794569452	0.099516918
TC1703829	1.79077824	0.007581085
LGMN	1.787702704	5.44E-07
GPR65	1.786931458	0.0734861
SLC25A24	1.779863873	0.0734861
IGH-VJ558	1.778496747	0.099516918
SLFN2	1.77554654	0.029429781
RGS2	1.774647029	0.014937438
P2RY6	1.77436975	0.001466487

UNC5B	1.773004383	0.003669217
GZMC	1.772449857	1.37E-05
SELPLG	1.771945144	1.71E-05
NGFRAP1	1.767167491	0.000774517
COL4A1	1.763388424	0.000167342
ITGAX	1.757531073	0.043182203
HMGCR	1.755655096	0.037937888
LIMD2	1.7541665	5.44E-07
PLSCR1	1.751436417	0.014937438
4933421I07RIK	1.74897612	0.000167342
A_55_P2093770	1.748784204	0.028990591
CLEC1B	1.744003775	0.000954713
SLC25A4	1.743228583	0.005147085
1810011O10RIK	1.74050435	0.002188424
ACTA2	1.736570298	0.043182203
ITPR3	1.7317125	1.71E-05
PTGR1	1.715605723	0.002188424
SMIM22	1.715533331	0.000189142
BC030867	1.711134571	0.059268219
ARHGAP9	1.706346787	3.24E-07
CLEC7A	1.70546921	0.005818331
RIPK3	1.704888253	0.000107205
C1QA	1.704655911	0.000107205
ALOX5AP	1.702608924	1.37E-05
MS4A6C	1.700017264	0.007581085
EAR10	1.697538919	0.000506785
AF251705	1.697450593	0.000107205
AKR1B8	1.696527005	0.029429781
CHR3:146629425-146649125_F	1.695523256	0.005147085
SRGN	1.69488308	0.002188424
HIST1H2AB	1.694816315	0.005818331
SCIMP	1.694232856	6.75E-05
ELL3	1.693514265	0.023459349
TUBB6	1.690673341	5.44E-07
TTK	1.685508494	0.059268219
EBI3	1.68123825	2.93E-07
CD9	1.67984899	3.73E-05
ENSMUST00000110087	1.677204304	0.037937888
THEM6	1.675583586	0.099516918
RNASE2A	1.674560725	0.000226234
GM7480	1.674247325	0.0734861
ORM1	1.673169342	0.059268219
SLC25A12	1.672923492	0.024306258
TNFAIP8L2	1.672668698	0.012257839
PITPNM1	1.671508995	0.000954713
ENSMUST00000118061	1.670856947	0.001324416
CD48	1.670419445	1.71E-05
HIST1H1B	1.669297844	0.005818331
GNG2	1.668879196	0.000954713

HTR1D	1.667817124	7.50E-05
AI429363	1.66657864	0.002188424
MAP4K4	1.664129418	1.77E-07
1700024P16RIK	1.661510302	0.011102126
KIF21B	1.653918907	0.005147085
EAR12	1.651625285	0.002188424
INPP5D	1.648096795	1.71E-05
PIRB	1.639279194	0.008029527
TNFAIP8	1.637395283	0.023459349
PROCR	1.636093223	0.059268219
MFSD7A	1.635846138	0.005818331
OLFML3	1.635695302	0.005818331
APAF1	1.634565689	0.003669217
EAR2	1.630587939	0.003669217
TSPAN6	1.628591958	0.0734861
CD68	1.624909437	6.75E-05
EVI2A	1.621337631	0.000506785
SLC4A8	1.618791055	0.024803337
MMP7	1.617007932	0.000189142
GMPR	1.615291841	0.043182203
PIF1	1.612916975	0.005818331
FAM102B	1.612662194	0.001466487
SULT2B1	1.61164897	0.0734861
HIP1	1.60425018	0.099516918
LBH	1.604249713	0.004840579
THEMIS2	1.603258305	0.009923494
PRMT2	1.602641935	0.011102126
HIST1H2AO	1.600204175	0.099516918
PCDH17	1.597815431	0.000774517
CHR17:12990484-13018375_R	1.59769806	0.089012317
AK7	1.597541526	0.0734861
KCNK6	1.593720086	0.000166554
HIST1H2AN	1.590515454	0.009923494
EMP3	1.59039437	0.014937438
ENSMUST00000093911	1.589731403	0.024306258
ARMCX4	1.587019989	0.000774517
IL17RB	1.583799238	7.10E-05
LY86	1.580465899	6.75E-05
FILIP1L	1.575596776	0.000167342
GM5640	1.575501842	0.005147085
PIK3CD	1.57311208	0.003229661
AKR1B10	1.573085365	6.76E-06
P2RY12	1.567058881	0.099516918
UST	1.564011401	0.023459349
IFI30	1.561488645	0.000346571
CLEC10A	1.56127431	0.099516918
CSTB	1.561018112	0.000954713
LYL1	1.559512621	0.012257839
HAUS8	1.559031333	0.0734861

PEA15A	1.555659226	0.000954713
PARVG	1.555073158	0.005818331
COL1A2	1.5524121	0.004640056
AOC1	1.551824344	0.003229661
HIST1H2AG	1.551696565	0.053870316
FCGR4	1.55047245	2.68E-05
CORO2A	1.54788136	0.014937438
GM10791	1.546119111	0.099516918
ENSMUST00000169692	1.53883719	0.024306258
AKAP3	1.538215661	0.000506785
ECSCR	1.538074638	0.002188424
SYNE4	1.537441003	0.043182203
SYCE2	1.53683133	0.043182203
CYTH4	1.536529385	0.002188424
A_55_P2071846	1.535950239	0.002188424
FPR1	1.535679989	0.000107205
ARHGAP44	1.532803698	0.001466487
SGCE	1.527873253	0.000189142
PKM	1.526685351	0.008029527
HECW2	1.520788055	0.024306258
GM10277	1.518957663	0.009923494
FKBP10	1.518656861	0.014937438
CXCL10	1.517942414	0.043182203
MBD4	1.51702753	0.011102126
SH3BGRL3	1.515850303	0.002188424
ITM2C	1.514009751	0.005147085
C1QC	1.51394394	1.71E-05
CALHM2	1.513046173	5.44E-06
ETS2	1.508735392	1.37E-05
SPAG5	1.507852211	0.014937438
LAMA5	1.506188522	7.10E-05
SSBP4	1.505530483	0.002188424
MYL12A	1.505396975	0.024306258
DDAH1	1.504267489	0.003669217
TSPAN13	1.50299822	0.011102126
PLXDC2	1.501863994	0.059268219
MPEG1	1.500480031	0.007581085
CLEC4A1	1.500062381	0.004840579
GDPD1	1.499706842	0.003669217
RRM1	1.496413676	0.00216544
HUNK	1.492265889	0.014937438
CLIP2	1.4905006	0.023459349
4933403J19RIK	1.488919063	0.0734861
PLD4	1.487706334	6.75E-05
CHR6:116123643-116124550_F	1.487259534	0.0734861
RHBDF1	1.485270523	0.014937438
CKB	1.484150064	0.000954713
PPAP2C	1.48207281	0.005818331
VAT1	1.47953232	0.005147085

EFCAB10	1.479467899	0.089012317
MOB3A	1.478690425	0.099516918
TTC39A	1.477948376	0.029429781
GLIS2	1.475857787	0.001466487
GUSB	1.475840868	0.003229661
AU015723	1.474519037	0.014937438
RASGRP1	1.472775001	0.059268219
ARHGAP25	1.472317995	0.002188424
CTSC	1.472051801	0.043182203
NAMPT	1.470683452	0.003669217
MMGT2	1.470321261	0.004840579
E2F1	1.466824367	7.10E-05
RFTN1	1.466778821	0.028990591
CD300E	1.465323349	0.059268219
EPPK1	1.464443829	0.014937438
CHR1:175653557-175671940_R	1.46210798	0.000506785
TMEM41A	1.459525075	0.005818331
TLR4	1.459306562	0.004840579
FEN1	1.458864755	0.024306258
PIRA2	1.457077034	0.037937888
ENSMUST00000195502	1.456279327	0.0734861
SIRPA	1.456091059	0.000167342
2700094K13RIK	1.456016274	0.099516918
PCNA	1.446851596	0.043182203
MATN2	1.445221219	0.0734861
LXN	1.444514956	0.005147085
VIM	1.444391449	0.014937438
HACD4	1.44438037	0.000954713
GPR137B	1.443865438	0.009923494
FCGR2B	1.44362754	0.011102126
RBL1	1.439341719	0.005818331
CHST11	1.438743127	0.059268219
CHR1:175654606-175672031_R	1.438240135	8.57E-06
AXL	1.437110478	0.000506785
PLEK	1.435541848	0.029429781
IGSF9	1.434626606	0.005818331
PLEKHB2	1.431689987	0.099516918
RASGEF1B	1.431578546	0.043182203
SULT1A1	1.430239061	0.002188424
PRELID2	1.427345877	0.024306258
TAX1BP3	1.42662508	0.001324416
RNF32	1.424058333	0.037937888
PTPRC	1.422998988	0.000954713
FAM174B	1.421514758	0.009923494
FCER1G	1.418943193	0.012257839
GM14446	1.418024647	6.75E-05
LIG1	1.415503962	0.003229661
MEST	1.413745921	0.099516918
LSP1	1.412438602	0.011102126

NCKAP1L	1.411757915	0.000346571
LOX	1.40916765	0.024306258
CCL24	1.408996678	0.059268219
A_55_P2036932	1.407184735	0.028990591
CIB3	1.406826312	0.099516918
5730508B09RIK	1.40617262	0.079872805
BATF	1.402589728	0.099516918
GADD45B	1.400971008	0.0734861
BC028528	1.399434905	0.000954713
FSTL1	1.397796455	0.001466487
TRIM47	1.391136801	0.002188424
A_55_P2032912	1.390405586	0.028990591
HPCAL1	1.386904631	0.000189142
LOXL2	1.384244255	0.099516918
PRRX1	1.38321652	0.059268219
GATA6	1.382799092	0.043182203
ENSMUST00000153890	1.382143011	0.014937438
A_55_P2170105	1.381956396	0.0734861
NFKBID	1.381031878	0.059268219
ALDH3B1	1.381015617	0.000954713
CHRX:98989930-98994344_F	1.380682255	0.000954713
OXCT1	1.380575466	0.005147085
RAC2	1.380024408	0.024306258
CCDC88B	1.379717306	0.029429781
GIT2	1.374364923	0.099516918
NFKBIE	1.366025145	0.024306258
TGFBI	1.365342815	0.001538172
CSRP1	1.364180493	0.009923494
WIPF3	1.362528889	0.002188424
NRROS	1.362064022	3.73E-05
ADAP1	1.361501379	0.000954713
LPXN	1.360637136	0.089012317
KCNU1	1.357957332	0.053870316
HPSE	1.356121768	0.005818331
MPZL1	1.354595127	0.007581085
APBB1IP	1.353781703	0.002188424
IGSF6	1.353084205	0.002188424
ADAM23	1.350778677	0.014937438
A_55_P2144126	1.348899179	0.007581085
MYLIP	1.34804234	0.000506785
GIMAP5	1.347815236	0.003229661
IFI204	1.346615188	0.000346571
MARCKS	1.346245838	0.002188424
COL6A3	1.34467492	0.099516918
LAIR1	1.34190092	0.002188424
GNGT2	1.339888764	0.023459349
RAB8B	1.33949339	0.014937438
IL10RA	1.339113816	0.002188424
SEMA6D	1.337583704	0.053870316

DBNL	1.335358811	0.043182203
PLCG2	1.333460585	0.002188424
HCK	1.331107314	0.003669217
KRTAP9-3	1.328513716	0.059268219
MAN1C1	1.325963954	0.009923494
CCL9	1.325803017	0.011102126
TLR1	1.323863672	0.0734861
PDGFA	1.323185419	0.043182203
REM2	1.322131738	0.099516918
GAS2L3	1.321756039	0.024306258
TGFBR2	1.321543879	0.001538172
FLNA	1.32142193	0.007581085
CTBP2	1.321303386	0.024306258
IFI205	1.318069717	0.024306258
CASP4	1.317360218	0.049978137
FOXD2OS	1.313272783	0.002188424
DEGS1	1.312872133	0.001466487
ADCY7	1.311565477	3.84E-05
COL4A2	1.30959008	0.009923494
CYP4F16	1.307444702	0.043182203
TLR13	1.306779444	0.024306258
STEAP2	1.306125114	0.099516918
KCTD12	1.300811298	0.037937888
A_55_P2040810	1.299158748	0.079872805
RUNDC3B	1.298903226	0.024306258
ADCK4	1.29719326	0.000774517
ENSMUST00000091117	1.296371333	0.005818331
WAS	1.289849798	0.002188424
TMSB10	1.28871503	0.037937888
SLC43A2	1.286729369	0.000107205
TUBA8	1.285518578	0.052789646
TMSB4X	1.284228922	0.023459349
TC1682680	1.283330453	0.037937888
LAPTM5	1.281336644	0.029429781
CCNJL	1.27921795	0.0734861
NCK2	1.277366159	0.000954713
ABLIM1	1.276439083	0.0734861
COL5A1	1.276069287	0.002188424
FCNA	1.275539358	0.0734861
ARHGAP18	1.273584194	0.079872805
SPCS1	1.271153829	0.099516918
HCLS1	1.270124578	0.004840579
SHISA5	1.265605483	0.059268219
IQGAP1	1.254519769	0.000506785
PDLIM4	1.25290363	0.009923494
DOCK11	1.252482963	0.024306258
ARF6	1.250301202	0.007581085
HIST1H1A	1.247997475	0.029429781
MAT1A	1.243517491	0.005818331

CD52	1.241606867	0.0734861
PTPN18	1.241313899	0.023459349
SNRPB	1.239392853	0.024306258
CRIP2	1.239007751	0.000107205
LRRC25	1.238898592	0.001324416
PCBP3	1.238056383	0.005818331
CTSK	1.236972778	0.052789646
SCAMP5	1.236172093	0.014937438
A_55_P1975843	1.235947776	0.059268219
PPIC	1.23505509	0.007581085
IFIT2	1.233711616	0.000162979
KANK3	1.232275384	0.029429781
NUP93	1.225725019	0.014937438
MUC4	1.223727674	0.099516918
FGR	1.223430155	0.043182203
HK3	1.222807934	0.000774517
YWHAH	1.222379889	0.005818331
DOK2	1.219200533	0.012257839
FABP4	1.218556279	0.08222578
PGK1	1.217288726	0.0734861
ARHGAP31	1.216954214	0.000954713
GM5643	1.216776956	0.043182203
TFPI	1.216258204	0.0734861
FES	1.215853865	0.099516918
ENSMUST00000155076	1.214388269	0.000346571
AI607873	1.211828423	0.043182203
EVL	1.210585771	0.043182203
NFAM1	1.209921235	0.005818331
SDC1	1.209717284	0.0734861
RGS10	1.208961328	0.059268219
SDPR	1.205065011	0.024306258
NPC2	1.204774545	0.059268219
2210013O21RIK	1.204659915	0.002188424
MAPK3	1.203777767	0.001466487
IRF8	1.19900585	0.003669217
AJAP1	1.197521556	0.099516918
ITGB2	1.196566353	0.089012317
APOC2	1.196503332	0.002188424
SNX20	1.195132916	0.024306258
RCN1	1.191525015	0.043182203
PCDHB6	1.190785671	0.005147085
KRT18	1.190322929	0.043182203
PAQR4	1.190112703	0.037937888
TPI1	1.18954168	0.005818331
C5AR2	1.188567157	0.089012317
RIPK4	1.188301049	0.004640056
ENSMUST00000155820	1.187731877	0.007581085
A_55_P2075000	1.18699201	0.029429781
SLC7A8	1.185100696	0.043182203

TYMS-PS	1.181782521	0.059268219
MYO1G	1.180747858	0.014937438
NAP111445-1	1.179973657	0.007581085
TSPAN17	1.179902522	0.014937438
TUBB4A	1.176785841	0.029429781
TIE1	1.176606404	0.099516918
HCST	1.176226744	0.079872805
CHR4:136856064-136877219_R	1.173400165	0.024306258
SMC4	1.172914335	0.005818331
BC030479	1.171947767	0.029429781
FLT4	1.169936253	0.009923494
AIFM2	1.168886504	0.099516918
CSF2RA	1.165507753	0.028990591
A_55_P2112302	1.164406618	0.005818331
SLC25A48	1.163896327	0.059268219
FAM129A	1.161384183	0.043182203
PDLIM1	1.159942444	0.053870316
PIGP	1.15777819	0.0734861
ANK3	1.156786935	0.079872805
VCPKMT	1.155105894	0.007581085
NAAA	1.154357795	0.043182203
RARG	1.152267696	0.099516918
CLCA3A1	1.152180427	0.002188424
CHRX:50407969-50410370_R	1.147428564	0.014937438
KIFC3	1.145783555	0.079872805
RDH5	1.142007346	0.014937438
ATP6V0E	1.141490493	0.059268219
SOX4	1.139588738	0.015085602
MRC1	1.138651938	0.059268219
ADAM15	1.137710105	0.099516918
VSIG4	1.137678972	0.0734861
PDLIM2	1.137224191	0.089012317
CHR8:119739165-119739905_F	1.133001437	0.0734861
KLF2	1.130396241	0.099516918
HIST1H2AK	1.129852911	0.024306258
TM6SF1	1.126130126	0.024306258
FANCD2	1.12399299	0.099516918
DPYSL2	1.123365197	0.005818331
PCOLCE	1.121930287	0.099516918
DENND3	1.121874386	0.007581085
NUDT19	1.121843412	0.009923494
PRKCA	1.120510616	0.015085602
MCL1	1.115832219	0.012257839
ARHGEF16	1.115004364	0.00216544
KPNA2	1.114960506	0.005818331
AW011956	1.113953515	0.005818331
ABI3	1.112316276	0.043182203
RNF169	1.112284796	0.0734861
GDE1	1.112128451	0.099516918

POLD1	1.109883471	0.037937888
GM4120	1.104073205	0.005818331
CAV2	1.104002851	0.043182203
CNN2	1.102042544	0.0734861
C030046G05	1.101455572	0.037937888
TMEM165	1.101210961	0.059268219
COL4A5	1.101189795	0.079872805
HIST2H3B	1.100668861	0.037937888
SIVA1	1.097391364	0.007581085
TEAD2	1.0971577	0.059268219
POC1A	1.096390272	0.029429781
ENSMUST00000172583	1.094851044	0.043182203
BQ042754	1.09378655	0.004640056
ARPC1B	1.089132773	0.0734861
CPM	1.086539072	0.059268219
POR	1.085874089	0.043182203
FAM111A	1.083782572	0.043182203
HIST2H2BB	1.083724155	0.024306258
PFKL	1.082426502	0.024306258
FPR2	1.080366922	0.059268219
HDAC7	1.080353878	0.099516918
MYO1D	1.079235952	0.099516918
BTNL9	1.078241365	0.023459349
LARGE	1.077550509	0.079872805
F11R	1.076535669	0.043182203
FANCB	1.075997398	0.029429781
VARS	1.07593971	0.079872805
MAP3K1	1.074745552	0.014937438
P2RY13	1.066582012	0.0734861
ADD3	1.065155445	0.053870316
RRAS	1.0641195	0.043182203
CHR16:34524589-34573439_R	1.063871582	0.099516918
TUBA1B	1.062086613	0.004840579
MORC3	1.061641417	0.0734861
ATP7A	1.061016511	0.005818331
CHR9:34195675-34196336_R	1.060662591	0.029429781
NAP096950-001	1.059772656	0.002188424
A_55_P2067483	1.053832367	0.037937888
IDH2	1.050184935	0.023459349
VOPP1	1.049388658	0.003669217
TMEM54	1.04895819	0.005147085
PYCARD	1.048543886	0.037937888
AKR1B3	1.045848862	0.014937438
FRY	1.045508926	0.0734861
TMEM120A	1.044075428	0.0734861
PDLIM7	1.042872283	0.0734861
CTSD	1.042696288	0.005818331
WDR1	1.041914685	0.031528574
NAP102538-1	1.038601595	0.037937888

HEXA	1.033694472	0.099516918
CCDC102A	1.030840179	0.029429781
ENSMUST00000019386	1.029372063	0.011102126
SLC1A5	1.024514605	0.003229661
RAB19	1.021950294	0.003229661
TONSL	1.016230778	0.099516918
TMEM132A	1.015648615	0.049978137
CDC42EP5	1.014981682	0.0734861
SAMM50	1.013730678	0.037937888
SNX12	1.012120535	0.024306258
ILF3	1.010048615	0.0734861
WWTR1	1.009861007	0.0734861
MIS18A	1.006911835	0.037937888
TGFB1	1.006877153	0.029429781
SLAMF9	1.004560788	0.099516918
MAPK7	1.003138254	0.043182203
H2AFX	0.999053844	0.0734861
G2E3	0.998894187	0.099516918
CDKN2D	0.992365693	0.059268219
SEMA4B	0.991360702	0.024306258
HSPG2	0.991099645	0.007581085
ARHGEF6	0.990092873	0.059268219
A_55_P2013591	0.988489354	0.079872805
CRTAP	0.988369674	0.037937888
FYB	0.985552492	0.052789646
NCF2	0.985116159	0.099516918
RALGDS	0.982851305	0.011102126
MVB12B	0.978261984	0.029429781
SOX18	0.978091538	0.079872805
GNB1	0.975514867	0.011102126
SMPDL3A	0.974359196	0.024306258
MARCKSL1	0.972043358	0.029429781
CSF1R	0.969373255	0.053870316
GTF2E2	0.968474903	0.059268219
ENSMUST00000181978	0.967115392	0.053870316
ARHGEF2	0.96473494	0.053870316
PRKAR1B	0.9535829	0.099516918
TC1615277	0.953483802	0.099516918
PYHIN1	0.952996272	0.049978137
HEXB	0.949823911	0.099516918
CALM2	0.949363373	0.014937438
EHBP1L1	0.949271539	0.059268219
SFXN1	0.949106721	0.005818331
USP12	0.948569133	0.043182203
HRH1	0.948183016	0.0734861
A_55_P1995151	0.946119423	0.079872805
ENSMUST00000044113	0.946093011	0.029429781
COLGALT1	0.945877455	0.089012317
ATPIF1	0.943333182	0.0734861

MKRN2OS	0.939128462	0.024306258
STARD10	0.937443151	0.059268219
ITIH3	0.93271268	0.0734861
YKT6	0.932506377	0.099516918
PLA2G15	0.931241418	0.0734861
MAGEF1	0.929944907	0.079872805
ITGA7	0.929788871	0.0734861
RAB3IL1	0.929624809	0.099516918
A_55_P2029021	0.929059293	0.024306258
ENSMUST00000182630	0.928552055	0.0734861
ENKD1	0.922473849	0.029429781
ENSMUST00000181990	0.920000856	0.099516918
CDC42SE1	0.91160615	0.059268219
A_55_P2108424	0.910059396	0.099516918
UNC13B	0.909376101	0.099516918
SLC15A4	0.906192027	0.099516918
TM4SF1	0.899023548	0.0734861
EFTUD2	0.891708959	0.0734861
TSPO	0.890264736	0.037937888
EMP2	0.887695229	0.0734861
GPX4	0.885927671	0.0734861
A930028N01RIK	0.879633198	0.099516918
A_55_P2153141	0.879292566	0.0734861
SLBP	0.879236483	0.043182203
SCARB1	0.875665435	0.099516918
TNIP1	0.874481936	0.099516918
UPP1	0.874264306	0.053870316
F2R	0.862581484	0.037937888
CORO1C	0.862417008	0.059268219
ENSMUST00000122262	0.861668141	0.099516918
CD99	0.860830633	0.0734861
MYL12B	0.859884072	0.099516918
LEPROT	0.846460043	0.053870316
RASA3	0.844646747	0.089012317
BCKDHB	0.842811174	0.037937888
A_55_P2072908	0.841065127	0.079872805
RANGAP1	0.838795528	0.099516918
ENSMUST00000067230	0.837008115	0.099516918
PGAM1	0.819396908	0.099516918
PYCR2	0.818495577	0.053870316
CTSB	0.814065532	0.099516918
CASP2	0.812082612	0.099516918
2810025M15RIK	0.79732462	0.0734861
ENO1B	0.796050435	0.0734861
ADAMTS2	0.793025174	0.0734861
CXCL16	0.779078519	0.099516918
A_55_P1963878	0.778911999	0.099516918
RAP1GAP	0.77829128	0.099516918
INPP5A	0.772764088	0.099516918

ATP11C	−0.734370962	0.099516918
NR3C1	−0.741071277	0.099516918
SCAF1	−0.777483758	0.08222578
CHR1:45066850-45095650_R	−0.789596549	0.099516918
NANP	−0.791336466	0.0734861
ARL5B	−0.804703013	0.099516918
MAU2	−0.812274334	0.099516918
TMEM220	−0.815890663	0.099516918
ZFP110	−0.820299486	0.059268219
TPGS1	−0.821327654	0.099516918
MXD4	−0.824900339	0.0734861
GM15517	−0.82840673	0.0734861
SARS	−0.830805522	0.089012317
NOG	−0.836295015	0.08222578
BTD	−0.837256181	0.099516918
CPOX	−0.84838691	0.099516918
ENSMUST00000118343	−0.849675239	0.087334443
2310010J17RIK	−0.851340893	0.099516918
CHMP7	−0.852217039	0.059268219
UBL7	−0.85263828	0.099516918
ZFP62	−0.853446907	0.079872805
A_55_P2128042	−0.855883408	0.099516918
CHR8:27129927-27137444_R	−0.85650172	0.099516918
RIBC1	−0.857335888	0.043182203
TC1630414	−0.857658144	0.059268219
FERMT2	−0.862585771	0.059268219
UQCC3	−0.867700805	0.08222578
CHR18:32389815-32392082_F	−0.868366745	0.099516918
VMN1R48	−0.871175936	0.087334443
A_55_P2114034	−0.87467089	0.0734861
AW111846	−0.876422996	0.059268219
GM29735	−0.878193252	0.099516918
CLPP	−0.878402679	0.023459349
ENSMUST00000051301	−0.878881248	0.0734861
LHFPL4	−0.88326082	0.0734861
TRP53INP2	−0.887697144	0.089012317
DICER1	−0.888844284	0.079872805
CHR16:43623175-43624069_F	−0.893172533	0.079872805
CHR14:33895294-33923644_F	−0.897525766	0.0734861
TRIP4	−0.898516551	0.029429781
C8A	−0.900000694	0.0734861
GM14432	−0.900028484	0.059268219
TC1639827	−0.900586524	0.0734861
NRSN2	−0.904226064	0.099516918
FBXO36	−0.905095525	0.024306258
CD3E	−0.905481551	0.0734861
1500012F01RIK	−0.914382543	0.029429781
ACPP	−0.915879811	0.099516918
SLC33A1	−0.91653669	0.011102126

CAPN11	−0.916970798	0.024306258
WRAP73	−0.918738246	0.014937438
ARHGAP26	−0.919914822	0.0734861
PDF	−0.920797069	0.024306258
A_55_P2032849	−0.921409641	0.005818331
CHR10:87541696-87547421_R	−0.923225353	0.043182203
PEX11A	−0.923653321	0.0734861
DCDC2B	−0.925984204	0.014937438
MOCS1	−0.92657884	0.099516918
FAM188A	−0.926625521	0.029429781
GJB1	−0.928802449	0.0734861
TPRKB	−0.9295728	0.059268219
TSACC	−0.930299617	0.024306258
1700019O17RIK	−0.931335601	0.024306258
CHR3:9401052-9415202_R	−0.932581262	0.079872805
TLE1	−0.933984821	0.0734861
NUDT12	−0.936308769	0.059268219
C330006A16RIK	−0.936955917	0.037937888
CHR12:17888352-17907493_F	−0.939762566	0.099516918
HCRTR1	−0.945085768	0.007581085
ACAT1	−0.94856341	0.029429781
CHR12:81719382-81731865_F	−0.953482625	0.014937438
FAM126B	−0.956784115	0.043182203
GM38521	−0.957775613	0.079872805
NAP093892-001	−0.960139433	0.099516918
CIR1	−0.960592962	0.059268219
SLC38A10	−0.962444579	0.014937438
SETD1B	−0.963838254	0.037937888
ACOX3	−0.964779403	0.024306258
GM4470	−0.964889622	0.029429781
ERLEC1	−0.966978456	0.029429781
DLX3	−0.970611312	0.099516918
EFNA5	−0.970863724	0.0734861
WDR34	−0.971454732	0.043182203
MET	−0.976863179	0.014937438
1700024P12RIK	−0.97888967	0.024306258
DENND6B	−0.979443478	0.024306258
CHR15:78404840-78412046_R	−0.986297164	0.029429781
CHR15:102947390-102971082_F	−0.991934303	0.024306258
EPN3	−0.99533442	0.005147085
PHKG2	−0.997699909	0.053870316
AP4M1	−1.003650881	0.024306258
CES1B	−1.005995126	0.009923494
ENSMUST00000119830	−1.007961627	0.037937888
A_55_P2083149	−1.008728747	0.043182203
UGT2B38	−1.010810683	0.043182203
HSD3B6	−1.012570394	0.005818331
ENSMUST00000177892	−1.013387048	0.005147085
A_55_P2040773	−1.013927465	0.037937888

CYP46A1	−1.015479513	0.0734861
OLFR128	−1.017585619	0.002188424
ORMDL2	−1.018483657	0.099516918
HIPK4	−1.023010794	0.043182203
CHR7:134375931-134376644_R	−1.024175781	0.043182203
CYP4F13	−1.025561317	0.001466487
POLI	−1.03523186	0.0734861
HSD3B3	−1.037561789	0.059268219
LGALS8	−1.045257465	0.014937438
CYB5D1	−1.046969998	0.005147085
KLHDC7A	−1.050112078	0.053870316
GPC1	−1.050470369	0.024306258
0610011F06RIK	−1.053335285	0.029429781
A_55_P2018307	−1.055704757	0.014937438
CHR16:13468953-13474394_F	−1.056026085	0.099516918
GM4013	−1.056581965	0.014937438
TC1608815	−1.057994969	0.005818331
FOXN3	−1.059486872	0.024306258
DARS	−1.059607975	0.023459349
GGACT	−1.062151716	0.005818331
ESRP2	−1.062595062	0.023459349
ENSMUST00000119126	−1.064502253	0.059268219
PRR5	−1.065806709	0.0734861
DHDH	−1.066809238	0.099516918
A_55_P2010539	−1.067620243	0.037937888
ENSMUST00000179479	−1.069629834	0.087334443
KRTAP19-5	−1.073291707	0.014937438
HSD3B2	−1.074637102	0.028990591
LHX1OS	−1.079640847	0.037937888
GM32934	−1.082758986	0.005147085
DVL3	−1.082940712	0.003669217
PNLDC1	−1.085478128	0.059268219
MPV17L	−1.085839512	0.099516918
CHR1:71939741-71947891_F	−1.088096702	0.099516918
CHR10:87822148-87841825_R	−1.088729278	0.005818331
ENSMUST00000086909	−1.094650826	0.003669217
A_55_P2048660	−1.102113848	0.005818331
TMEM158	−1.102447413	0.009923494
PIGC	−1.102954085	0.011102126
MPDZ	−1.10857045	0.0734861
GM11213	−1.1096467	0.005147085
CMTM6	−1.110395571	0.011102126
GLUL	−1.111557528	0.0734861
CHR7:50509139-50526856_R	−1.113027381	0.0734861
A_55_P2168599	−1.116385668	0.043182203
ABHD17C	−1.122894418	0.023459349
AU020094	−1.124187225	0.024306258
FRMD8OS	−1.131404264	0.0734861
RNF103	−1.13140447	0.0734861

AOX3	−1.132559548	0.089012317
PQLC1	−1.134770761	0.037937888
NAP114395-1	−1.137056218	0.000954713
TC1637255	−1.137107429	0.009923494
TC1640761	−1.13780723	0.009923494
MUP-PS16	−1.137969404	0.024306258
2900072G11RIK	−1.137989485	0.023459349
CHR7:134369091-134376468_F	−1.139238176	0.005818331
ENSMUST00000155958	−1.141694481	0.0734861
AK2	−1.146312502	0.043182203
GRB14	−1.147385666	0.000954713
UPK1A	−1.151997811	0.024306258
HSF2	−1.153000531	0.000774517
1500004F05RIK	−1.153825579	0.004840579
4732419C18RIK	−1.154149417	0.099516918
MPST	−1.154868114	0.005818331
VMN1R63	−1.161615463	0.099516918
CARS	−1.163206051	0.059268219
N4BP2	−1.163477182	0.012257839
PPP1R16B	−1.163514041	0.0734861
SLC17A2	−1.166968603	0.024306258
ENSMUST00000117213	−1.168264997	0.014937438
AYM1	−1.169282061	0.002188424
KRTAP19-4	−1.171591129	0.001466487
DDO	−1.171998383	0.024306258
HAO1	−1.172888344	0.002188424
GM4951	−1.173219605	0.029429781
OLFR544	−1.174526181	0.007924415
ACSM1	−1.176159687	0.005818331
ELOVL2	−1.178354499	0.012257839
GM4881	−1.1791624	0.005818331
GM10216	−1.179364089	0.014937438
D630039A03RIK	−1.179763264	0.005818331
MGMT	−1.183633457	0.059268219
ENSMUST00000131638	−1.185757713	0.043182203
TMEM254A	−1.188154923	0.005147085
GPCPD1	−1.188420351	0.029429781
ENSMUST00000117580	−1.192377563	0.007581085
UTP14A	−1.197260308	0.079872805
COX8B	−1.197997593	0.089012317
SLC10A5	−1.204428218	0.037937888
SCN5A	−1.208412712	0.087334443
GHR	−1.213080408	0.009923494
SLC2A9	−1.213424068	0.002188424
C030009H01RIK	−1.218357989	0.000774517
ABCA8B	−1.222969237	0.001466487
GM9924	−1.224068489	0.053870316
Mrz 02	−1.227660163	0.099516918
COPS7B	−1.228051918	0.005147085

A_55_P2097972	−1.235317713	0.005818331
CHR5:124489025-124506750_F	−1.240017159	0.007581085
CHR9:65647428-65677119_R	−1.241822875	0.023459349
DMTF1	−1.244898776	0.0734861
A930009L07RIK	−1.25048597	0.079872805
PTK7	−1.252471627	0.011102126
1700022A21RIK	−1.252605672	0.000774517
WDR91	−1.257879974	0.028990591
LOC105243194	−1.261512364	0.024306258
CHR17:39983902-39985688_F	−1.263318786	0.037937888
LPO	−1.269552613	0.099516918
ENSMUST00000110832	−1.27657604	0.099516918
PURA	−1.278548454	0.029429781
NPR2	−1.287624533	0.000166554
FBXL20	−1.288357964	0.059268219
ERN1	−1.297023788	0.000346571
CYP2D11	−1.299373375	7.10E-05
ADCK5	−1.299533724	0.087334443
WHSC1L1	−1.300418415	0.002188424
PPARA	−1.301069261	0.029429781
ENSMUST00000068783	−1.302388902	0.014937438
ENSMUST00000114694	−1.306284879	0.029429781
ARHGAP32	−1.307676973	0.000774517
MUP20	−1.307888942	0.005147085
TCF24	−1.316840966	0.0734861
UGT2B5	−1.320298374	0.002188424
CYP2D34	−1.320668065	0.000346571
CCDC157	−1.329331047	0.0734861
SCYL3	−1.331729901	0.043182203
RBPJ	−1.334304981	0.000226234
A_55_P2144090	−1.337351955	0.005818331
VMN1R87	−1.340893501	0.005147085
ENSMUST00000142141	−1.34500646	0.0734861
NAP112345-1	−1.345552308	0.000107205
CHR10:81680941-81704964_R	−1.346845943	0.049978137
FSCN3	−1.347732356	1.71E-05
A_55_P2178388	−1.352991915	0.079872805
CYP2D9	−1.353579738	0.000506785
TC1670009	−1.357462366	0.023459349
SERPINA11	−1.360415533	0.043182203
SLC4A2	−1.361450407	6.75E-05
RTP3	−1.365394337	0.024306258
GNAT2	−1.367759118	0.003669217
GM7768	−1.369961101	0.0734861
NR1H4	−1.370886038	0.005147085
ADGRF1	−1.373906653	0.009923494
ENSMUST00000170486	−1.376296213	0.099516918
PBLD2	−1.383111254	0.043182203
A_55_P2116794	−1.386233653	0.000506785

FOXD3	−1.386803126	0.014937438
AI317395	−1.400969674	0.043182203
MS4A3	−1.402251196	0.099516918
TC1758295	−1.411633609	0.037937888
COMT	−1.412082353	0.005818331
UGT2B37	−1.416163965	0.000506785
CHR1:85304494-85305390_R	−1.419415145	3.84E-05
GM6252	−1.42351622	0.0734861
RHOX2E	−1.423844568	0.000107205
ENSMUST00000160443	−1.429098882	0.004840579
CHR18:35142575-35269099_F	−1.44683145	0.011102126
GRIK5	−1.448546108	0.000774517
SDR42E1	−1.450789733	0.005147085
ABLIM3	−1.45357556	0.014937438
TC1603266	−1.459474942	0.002188424
TTC39B	−1.465473628	0.059268219
INO80B	−1.471962429	0.023459349
C78653	−1.473233349	0.029429781
GM10387	−1.483378859	0.089012317
TESCL	−1.494155742	0.0734861
MESP2	−1.495717849	0.002188424
ENSMUST00000117241	−1.497507044	1.71E-05
TCF7L2	−1.50216804	0.053870316
MIA2	−1.507950534	0.023459349
A_55_P2110671	−1.522010802	0.0734861
PTK2B	−1.528116628	3.24E-07
A830052D11RIK	−1.529657109	0.053870316
TTC39C	−1.5325637	1.31E-06
HSBP1L1	−1.534193903	1.40E-06
MN1	−1.543814829	0.029429781
CHR9:41368823-41369514_F	−1.546235123	0.099516918
F11	−1.551677484	1.20E-06
PDCD4	−1.571922804	0.059268219
NAP017825-001	−1.574751594	0.005818331
SULT1B1	−1.580479927	0.007581085
GM10319	−1.597878568	0.099516918
RBBP8NL	−1.617695139	0.0734861
D130020L05RIK	−1.634778695	0.000189142
CYP2C44	−1.651348007	0.000506785
PCP4	−1.656206616	0.037937888
CHR5:137395007-137412976_F	−1.671432312	0.014937438
ADH4	−1.676165876	0.0734861
5830473C10RIK	−1.680080382	0.099516918
ARSG	−1.680520024	3.84E-05
ENSMUST00000006786	−1.696290337	0.003229661
IGF1OS	−1.700733092	0.012257839
SOHLH2	−1.701973869	0.009923494
ENSMUST00000178355	−1.703229861	0.059268219
BB759059	−1.708061952	0.024306258

HOPX	−1.714965065	3.50E-06
GM5106	−1.718248164	0.000346571
GM32743	−1.719782174	0.005818331
CCBL1	−1.729289087	0.000346571
MBL2	−1.730021705	0.0734861
A_55_P2055423	−1.735685626	0.053870316
ENSMUST00000156405	−1.736935994	0.002188424
MAPT	−1.74444083	1.85E-06
UGT3A2	−1.761274696	0.043182203
RNASE10	−1.762157381	0.029429781
9330151L19RIK	−1.762618962	0.004840579
ENSMUST00000178292	−1.76359256	0.001466487
RD3	−1.790340379	0.003669217
TBX3	−1.791334689	0.029429781
GPR162	−1.81038102	0.014937438
ENSMUST00000119215	−1.81313112	0.014937438
ENSMUST00000188378	−1.849210632	0.001466487
ACSL1	−1.849429656	0.023459349
CHR5:113771679-113771745_F	−1.870888487	0.014937438
RARRES1	−1.873071647	0.005818331
CLEC2E	−1.87657829	0.000954713
CYP2D37-PS	−1.882848989	0.014937438
B3GALT1	−1.883919189	0.059268219
2310047N11RIK	−1.890649479	0.029429781
ENSMUST00000166759	−1.892271503	0.007924415
SLCO2B1	−1.894342247	0.014937438
F830016B08RIK	−1.901167232	0.000506785
ENSMUST00000092891	−1.907744114	1.32E-07
AI463229	−1.919942786	6.76E-06
GM12992	−1.924421185	0.000774517
REC8	−1.92865837	0.005818331
GM10765	−1.934834104	1.45E-11
AK036131	−1.938354988	0.099516918
GM4673	−1.965690811	3.24E-07
ARRDC3	−1.967692956	0.011102126
A_55_P2045859	−1.974519216	7.02E-09
BCO2	−1.977644673	0.000107205
ALAS2	−1.99085944	0.099516918
CYP4F14	−2.000992542	0.004640056
CHR19:36699712-36763966_R	−2.01004913	0.014937438
MRGPRG	−2.010581277	0.014937438
SYT1	−2.017004043	2.93E-07
CXCL9	−2.018711434	0.037937888
FABP2	−2.052084919	0.004840579
CYP2J9	−2.061549821	3.84E-05
BDH2	−2.0746301	0.003669217
FAM183B	−2.079054749	0.002188424
ENSMUST00000028205	−2.082093006	0.037937888
NEB	−2.088614503	0.029429781

A_55_P2124361	−2.091005251	1.35E-13
A730091E23RIK	−2.097909594	0.079872805
SRD5A1	−2.111880014	1.37E-05
HSD17B2	−2.130912949	5.01E-08
MEP1B	−2.136805137	0.001466487
LEAP2	−2.172101736	0.0734861
GM6329	−2.209993804	1.80E-08
SULT2A7	−2.220379379	0.053870316
SERPINA3K	−2.25640947	1.71E-05
CHRNA2	−2.273732358	6.85E-07
CYP2D13	−2.277721358	3.24E-07
CMAH	−2.342498594	3.24E-07
CES1E	−2.363835114	0.005818331
GBP6	−2.396856406	0.014937438
IGF1	−2.402914748	1.35E-13
SCNN1A	−2.404642653	0.005818331
CYP2J5	−2.411382469	0.000346571
C8B	−2.430080327	1.35E-13
SLC17A8	−2.432659842	0.053870316
ENSMUST00000079597	−2.443126969	0.0734861
1700054M17RIK	−2.449681192	0.0734861
PPP1R3C	−2.456839628	0.024306258
1810008I18RIK	−2.481004715	0.0734861
NCMAP	−2.485925713	1.35E-13
ENSMUST00000179900	−2.514262792	6.85E-07
CEBPE	−2.528545887	1.71E-05
RS5-8S1	−2.54361983	0.043182203
TSPAN33	−2.563997001	1.35E-13
PAQR9	−2.583750465	0.011102126
KRT80	−2.589493921	1.37E-05
GM128	−2.618935985	7.10E-05
AK157043	−2.630704357	1.31E-06
TIAM2	−2.692593713	0.099516918
MUP-PS12	−2.692741763	0.000774517
5730414N17RIK	−2.698411338	3.84E-05
SERPINA3H	−2.707920948	2.81E-09
EGFR	−2.714474074	6.15E-07
UGT2A3	−2.716220262	5.44E-07
CHR15:99765243-99783300_F	−2.726867248	0.000346571
RIPPLY2	−2.734981179	0.004640056
PDILT	−2.738114204	1.35E-13
GM10556	−2.749978864	0.000107205
AQP4	−2.760360176	0.029429781
IGLON5	−2.770799967	5.44E-06
LAMA3	−2.78438832	0.000506785
CYP2C67	−2.823433355	3.06E-09
SOCS2	−2.915032471	4.86E-05
SLC13A2	−2.921418223	0.089012317
A_55_P2019989	−2.945547649	0.000774517

ENSMUST00000119998	−2.978686944	2.44E-09
EXTL1	−3.029211053	0.000167342
SDR9C7	−3.045165597	1.37E-05
LHX3	−3.061743753	0.005818331
MCM10	−3.063168841	6.76E-06
MUP3	−3.075321987	1.35E-13
NREP	−3.079776675	1.37E-05
MUP4	−3.10264112	1.35E-13
GNA14	−3.119396527	0.007581085
SLC22A28	−3.135592263	1.45E-11
NUDT7	−3.166999906	1.85E-06
PAX2	−3.172269525	1.31E-06
UGT2B1	−3.21278232	3.84E-05
CYP2C68	−3.214429804	3.39E-08
ENSMUST00000126217	−3.244938218	5.44E-07
A_55_P2153191	−3.249711767	0.001466487
ABCA8A	−3.250805497	6.85E-07
BC025829	−3.349585551	0.024306258
PROK1	−3.354149352	1.35E-13
ENSMUST00000121473	−3.428041087	0.043182203
ENSMUST00000073521	−3.643994154	0.000774517
ZAP70	−3.64509225	5.69E-06
SNTG2	−3.668118163	1.35E-13
INHBE	−3.673841445	3.84E-05
SERPINA12	−3.864739397	1.45E-11
1700016K05RIK	−3.872802363	1.35E-13
CSRP3	−3.875344883	0.002188424
A_55_P1952156	−3.924589273	3.39E-08
C730036E19RIK	−3.930395708	3.39E-08
DCT	−4.072246939	0.049978137
SUSD4	−4.217008895	6.15E-07
ENSMUST00000171077	−4.253122933	3.39E-08
MUP5	−4.388002112	1.35E-13
MUP1	−4.408016739	0.002188424
CYP7B1	−4.501747394	1.35E-13
SULT5A1	−4.513003678	2.44E-09
CML5	−4.519837469	0.001466487
CSPG5	−4.571665023	0.000107205
IGFALS	−4.674482341	1.32E-07
SLC22A30	−4.68994555	0.000506785
OLFR1535	−4.826866365	3.39E-08
ACSM2	−4.960168739	1.45E-11
CES1F	−4.977875238	0.001466487
CES4A	−5.251422438	1.35E-13
2810007J24RIK	−5.355062789	8.29E-11
OLFR125	−5.411922924	1.35E-13
LOC545966	−5.569226448	0.002188424
ENSMUST00000120662	−5.585602075	0.005818331
GM3734	−5.715481795	1.35E-13

KEG1	−5.825939868	2.18E-11
ENSMUST00000178663	−6.218434989	0.053870316
CLEC2H	−6.406953676	3.39E-08
MUP2	−6.563348897	0.043182203
SERPINA9	−6.646456569	1.35E-13
CES3A	−6.673014005	0.000189142
MUP21	−6.799687296	1.35E-13
ENSMUST00000154733	−7.208291889	4.45E-05
ENSMUST00000121741	−7.262909762	1.35E-13
MUP6	−7.304048719	1.35E-13
TC1687046	−7.561199041	0.000107205
CES3B	−7.791466993	3.24E-07
SLCO1A1	−7.835438272	1.35E-13
CYP4A12A	−8.427628064	1.35E-13
ELOVL3	−9.066127339	1.35E-13
SERPINA4-PS1	−9.745270049	1.35E-13
CYP4A12B	−9.906360031	1.35E-13
HSD3B5	−10.93628773	1.35E-13

Table represents differentially regulated genes. RNA was extracted whole liver tissue from *Rbpj*$^{+/+}$ and *Rbpj*$^{-/-}$ mice at the age of 4 weeks. Log2 fold change and false discovery rate is listed for each single gene (n = 3–4, Shrinkage T-test, fdr < 0.1).

Table S3. Regulation of Hippo pathway genes.

Gene	Log2 Fold Change	False Discovery Rate
SERPINE1	4.961996904	0.482212591
WNT10A	4.110943424	1.35E-13
BMP8B	3.071683723	0.011102126
AFP	2.934659184	6.15E-07
BIRC5	2.329030992	0.1350845
CTGF	2.261069356	0.345591697
ACTG1	1.972301746	0.000167342
CCND1	1.857258085	0.005147085
ID4	1.79610855	0.0734861
WNT9B	1.584973069	1
AJUBA	1.503185704	0.132349936
TGFBR2	1.321543879	0.001538172
MYC	1.288861505	1
WTIP	1.268670756	0.176355558
YWHAH	1.222379889	0.005818331
FZD3	1.220547374	1
ITGB2	1.196566353	0.089012317
MOB1A	1.196559574	1
TEAD4	1.176534934	1
TEAD1	1.104763387	0.345591697
TEAD2	1.0971577	0.059268219
AREG	1.073652426	1
BMP6	1.058613337	1

WWTR1	1.009861007	0.0734861
TGFB1	1.006877153	0.029429781
PARD6G	0.950287983	1
TGFB2	0.926845804	1
TGFB3	0.79943766	1
CTNNA3	0.773674256	1
YWHAQ	0.762777475	0.199556568
RASSF1	0.739434338	0.482212591
CSNK1E	0.730945389	1
PPP2CB	0.679223995	1
SMAD2	0.647855579	1
PPP1CB	0.617266098	1
CCND3	0.594634174	0.345591697
BIRC2	0.576553277	1
YWHAB	0.576212712	1
PPP2R1A	0.565919314	1
PPP1CC	0.539831583	1
CDH1	0.519201066	1
FBXW11	0.50747507	1
CTNNB1	0.501578632	1
INADL	0.494572662	1
CTNNA1	0.490333843	1
AMOT	0.470649671	1
SMAD1	0.457994387	1
BMP7	0.45400364	1
ITGB2L	0.415932961	1
YWHAZ	0.407391768	1
PPP2R2A	0.404829188	1
YWHAG	0.384962691	1
LATS2	0.370249957	1
SMAD3	0.367968934	1
CRB2	0.35041503	1
BMPR2	0.347813504	1
DLG4	0.339074595	1
FRMD6	0.328064169	1
PPP2R2B	0.327735468	1
PPP1CA	0.327252809	1
WNT4	0.323268751	1
DVL2	0.314914989	1
CCND2	0.309816069	1
WNT3	0.28929643	1
PPP2CA	0.277436433	1
SMAD4	0.240689517	1
WWC1	0.239226946	1
PPP2R2D	0.229525677	1
CRB1	0.226894667	1
ACTB	0.224646095	1
TGFBR1	0.212771303	1
AXIN1	0.208146439	1

GLI2	0.207863093	1
CSNK1D	0.198813092	1
TRP53BP2	0.194320567	1
PRKCI	0.190498694	1
WNT7A	0.174333711	1
NF2	0.165164653	1
CTNNA2	0.14315798	1
PARD6A	0.129413465	1
YWHAE	0.128349186	1
SAV1	0.126428978	1
WNT5B	0.118672115	1
FZD5	0.109693485	1
GSK3B	0.100610005	1
WNT11	0.099830393	1
NKD1	0.077278888	1
RASSF6	0.067144656	1
BTRC	0.064439621	1
GDF6	0.054720129	1
SMAD7	0.050913177	1
DLG2	0.050437738	1
PPP2R1B	0.037083912	1
PPP2R2C	0.03404266	1
ID3	0.033633461	1
WNT2B	0.01418438	1
BMP2	0.010499537	1
BMPR1A	−0.002060653	1
FZD10	−0.026662878	1
STK3	−0.029818479	1
FZD1	−0.032364003	1
SNAI2	−0.051875497	1
SCRIB	−0.053718284	1
TCF7L1	−0.054855001	1
GDF7	−0.082544032	1
AMH	−0.084143753	1
FZD2	−0.084743419	1
TCF7	−0.101957677	1
MPP5	−0.137134254	1
LIMD1	−0.137785049	1
DVL1	−0.138307415	1
ID1	−0.140511454	1
FZD6	−0.142051892	1
WNT16	−0.15437104	1
DLG1	−0.160160012	1
WNT3A	−0.160911247	1
LLGL1	−0.174908691	1
YAP1	−0.19584197	1
FZD4	−0.209718764	1
BMP5	−0.213036174	1
SOX2	−0.221273069	1

WNT5A	−0.23747125	1
WNT10B	−0.290286253	1
APC2	−0.294596009	1
LATS1	−0.294723469	1
WNT7B	−0.339633939	1
PARD6B	−0.35260349	1
APC	−0.353384327	1
PRKCZ	−0.358286142	1
LEF1	−0.362600671	1
WNT9A	−0.396819769	1
MOB1B	−0.423065576	1
LLGL2	−0.460543643	1
DLG3	−0.461184175	1
TEAD3	−0.484223174	1
PARD3	−0.533816082	1
WNT2	−0.537021563	1
TRP73	−0.538973665	1
AXIN2	−0.546825621	1
BMP4	−0.552484074	1
FZD7	−0.575967222	1
WNT1	−0.611269258	1
BMPR1B	−0.645831753	1
WNT8A	−0.673180729	1
BMP8A	−0.714266045	1
ID2	−0.726289451	1
GDF5	−0.77301589	1
WNT6	−0.803197414	1
BBC3	−0.857864811	1
WNT8B	−0.999313544	1
FGF1	−0.999992498	0.176355558
FZD9	−1.023702172	1
DVL3	−1.082940712	0.003669217
FZD8	−1.228532511	1
TCF7L2	−1.50216804	0.053870316

Table presents overview of log2 fold change expression (*Rbpj*$^{+/+}$ vs. *Rbpj*$^{-/-}$ mice) for all Hippo pathway genes (n = 3–4, Shrinkage T-test, fdr ≤ 1).

Table S4. Antibodies.

**Antibody**	**Company**	**Catalog Number**	**Dilution (IHC)**	**Dilutions (WB)**
Albumin	Bethyl	A90-135	1:100	-
CD45	BD Pharmingen	550539	1:200	-
Cytokeratin 7	Abcam	ab181598	1:300	-
Cytokeratin 19	Santa Cruz	sc-33111	1:250	-
F4/80	ThermoFischer Scientific	MA5-16624	1:200	-
HNF4α	ThermoFischer Scientific	K9218	1:200	-
IQGAP1	Santa Cruz	sc-376021	1:200	1:1000

PCNA	MerckMillipore(Calbiochem)	NA03	1:150	1:1000
RBPJ	Cosmo Bio	2ZRBP3	1:200	1:1000
SOX-9	Merck - Millipore	AB5535	1:500	-
YAP1	Cell Signaling	14074	1:200	-
YAP1	Cell Signaling	4912	-	1:1000
Lamin B1	Santa Cruz	374015	-	1:500

Table presents a list of used antibodies for IHC/IF and western blot.

Table S5. Primer.

**Gene**	**Forward Primer (5′-> 3′)**	**Reverse Primer (5′-> 3′)**
αSMA	TCACCATTGGAAACGAACG	ATAGGTGGTTTCGTGGATGC
Afp	AGTTTCCAGAACCTGCCGAG	CTGGAGGTTTCGGGATCCAA
Ankrd1	GCTGAACAAAGGAGCCAAA	ATAGCGGTTCAGCCTCACAG
CK19	GGACCCTCCCGAGATTACAACCA	GCCAGCTCCTCCTTCAGGCTCT
Col1a1	CATGTTCAGCTTTGTGGACCT	GCAGCTGACTTCAGGGATGT
Ctgf	GGGCCTCTTCTGCGATTTC	ATCCAGGCAAGTGCATTGGTA
Cyr61	CTGCGCTAAACAACTCAACGA	GCAGATCCCTTTCAGAGCGG
Dtx1	CCAACCCAGGCAAGAAGTT	GATGAGCAATCTCAGCACCTT
Gpc3	TGTCACCAGGTCCGTTCTTT	GTTGTTCCATGTTCAGCCGT
Hes1	TGCCAGCTGATATAATGGAGAA	CCATGATAGGCTTTGATGACTTT
Hey1	ACCATCGAGGTGGAAAAGG	CTTCTCGATGATGCCTCTCC
Itgb2	GTGGTGCAGCTCATCAAGAA	GCCATGACCTTTACCTGGAA
IQGAP1	GGCAGAACGTGGCTTATGA	AAGGCCCTCCTCTAGCTCTG
Notch1	CAATGTTCGAGGACCAGATGG	ACTGCAGGAGGCAATCATGAG
Notch2	TGCCTGTTTGACAACTTTGAGT	GTGGTCTGCACAGTATTTGTCAT
Notch3	AGCTGGGTCCTGAGGTGAT	AGACAGAGCCGGTTGTCAAT
Notch4	GGACCTGCTTGCAACCTTC	CCTCACAGAGCCTCCCTTC
Sox9	TGCCCATGCCCGTGCGCGTCAA	CGCTCCGCCTCCTCCACGAAGGG
Tead2	GGAAGGCAGCGAAGAGGGC	CTTCACGTCTGGAACATTCCAT
YAP	CCTGATGATGTACCACTGCC	GCCATGTTGTTGTCTGATCG
RNA polymerase II	CATCAACCAGGTGGTACAGC	GATTCTGGAACTCAACACTCTCC
Rbpj	Mm_Rbpj_1_SG QuantiTect Primer Assay (QT00142821)	

Table presents primer and primer sequences used for qRT-PCR.
